# Efficacy and Safety of AmBisome in Combination with Sodium Stibogluconate or Miltefosine and Miltefosine Monotherapy for African Visceral Leishmaniasis: Phase II Randomized Trial

**DOI:** 10.1371/journal.pntd.0004880

**Published:** 2016-09-14

**Authors:** Monique Wasunna, Simon Njenga, Manica Balasegaram, Neal Alexander, Raymond Omollo, Tansy Edwards, Thomas P. C. Dorlo, Brima Musa, Mohammed Hassan Sharaf Ali, Mohammed Yasein Elamin, George Kirigi, Rashid Juma, Anke E. Kip, Gerard J. Schoone, Asrat Hailu, Joseph Olobo, Sally Ellis, Robert Kimutai, Susan Wells, Eltahir Awad Gasim Khalil, Nathalie Strub Wourgaft, Fabiana Alves, Ahmed Musa

**Affiliations:** 1 Drugs for Neglected Diseases *initiative*, Nairobi, Kenya; 2 Centre for Clinical Research, Kenya Medical Research Institute, Nairobi, Kenya; 3 Drugs for Neglected Diseases *initiative*, Geneva, Switzerland; 4 MRC Tropical Epidemiology Group, London School of Hygiene & Tropical Medicine, London, United Kingdom; 5 Department of Pharmacoepidemiology and Clinical Pharmacology, Utrecht Institute of Pharmaceutical Sciences, Faculty of Science, Utrecht University, Utrecht, the Netherlands; 6 Institute of Endemic Diseases, University of Khartoum, Khartoum, Sudan; 7 Department of Pharmacy & Pharmacology, Antoni van Leeuwenhoek Hospital, Amsterdam, the Netherlands; 8 KIT Biomedical Research, Amsterdam, the Netherlands; 9 College of Health Sciences, Addis Ababa University, Addis Ababa, Ethiopia; 10 Department of Medical Microbiology, Leishmaniasis Unit, College of Health Sciences, Makerere University, Kampala, Uganda; Institute of Tropical Medicine, BELGIUM

## Abstract

**Background:**

SSG&PM over 17 days is recommended as first line treatment for visceral leishmaniasis in eastern Africa, but is painful and requires hospitalization. Combination regimens including AmBisome and miltefosine are safe and effective in India, but there are no published data from trials of combination therapies including these drugs from Africa.

**Methods:**

A phase II open-label, non-comparative randomized trial was conducted in Sudan and Kenya to evaluate the efficacy and safety of three treatment regimens: 10 mg/kg single dose AmBisome plus 10 days of SSG (20 mg/kg/day), 10 mg/kg single dose AmBisome plus 10 days of miltefosine (2.5mg/kg/day) and miltefosine alone (2.5 mg/kg/day for 28 days). The primary endpoint was initial parasitological cure at Day 28, and secondary endpoints included definitive cure at Day 210, and pharmacokinetic (miltefosine) and pharmacodynamic assessments.

**Results:**

In sequential analyses with 49–51 patients per arm, initial cure was 85% (95% CI: 73–92) in all arms. At D210, definitive cure was 87% (95% CI: 77–97) for AmBisome + SSG, 77% (95% CI 64–90) for AmBisome + miltefosine and 72% (95% CI 60–85) for miltefosine alone, with lower efficacy in younger patients, who weigh less. Miltefosine pharmacokinetic data indicated under-exposure in children compared to adults.

**Conclusion:**

No major safety concerns were identified, but point estimates of definitive cure were less than 90% for each regimen so none will be evaluated in Phase III trials in their current form. Allometric dosing of miltefosine in children needs to be evaluated.

**Trial Registration:**

The study was registered with ClinicalTrials.gov, number NCT01067443

## Author Summary

Visceral leishmaniasis, or kala-azar, is a parasitic disease which is fatal without treatment. A 17-day treatment of sodium stibogluconate (SSG) with paromomycin (PM) is the recommended treatment in eastern Africa, but requires painful injections, causes adverse events, and patients need to stay in the hospital during treatment. An affordable, safe and effective oral treatment would be preferable. Whilst research to identify entirely new drugs is underway, existing treatments are being optimized as a short-term solution. Combination regimens based on AmBisome and miltefosine have been shown to be safe and effective in treating Indian patients, but there are no published data from use of these drugs in combination regimens from Africa, where efficacy of treatments can be different from India. Three regimens were evaluated for treating VL in eastern Africa, using AmBisome in combination with SSG or miltefosine, or miltefosine alone. Once again, drugs which are effective in India were found to be less so in African patients, and none of the regimes tested showed sufficiently high definitive cure rates to evaluate in Phase III trials. The results also suggest miltefosine was under-dosed in children and so allometric dosing, which takes into account the differences in drug metabolism seen in children compared to adults, needs to be studied.

## Introduction

Visceral leishmaniasis (VL), caused by *L*. *donovani*, is a neglected disease in eastern Africa, where it affects mostly the very poor. The disease burden is high, with an estimated 29,400 to 56,600 cases annually[1]. VL is fatal if left untreated, but with access to early diagnosis and treatment the case fatality rate is low[2].

For decades, treatment with pentavalent antimonials (sodium stibogluconate-SSG) was the first-line regimen in Africa despite a risk of cardiotoxicity, liver and pancreatic toxicity and 4 weeks of hospitalization [3]. A 17-day regimen of SSG in combination with paromomycin (PM) demonstrated high efficacy six months post end of treatment in a Phase III trial (91%) and is recommended as first line treatment in the eastern African countries, Sudan, South Sudan, Kenya, Uganda, Ethiopia, and Somalia [4,5]. Still, this short-course treatment is considered suboptimal in terms of route of administration, duration of treatment and potential adverse drug reactions, and there is a need for new treatments.

An oral, safe, inexpensive, highly effective, short course treatment suitable for use in pregnancy is also urgently needed to control VL in the region [6], and research is currently underway to identify and evaluate potential new candidates. In the meantime, as a short-term strategy, it was considered relevant to assess the efficacy of combination therapy with currently available treatment options. Combination regimens are generally recommended on the basis of potential additive or synergistic activity, increasing efficacy and allowing for shorter course durations, in turn leading to increased compliance, shorter hospitalization and reduced drug costs [6]. The rationale on the choice of drugs to combine is described below.

A short course of AmBisome (5mg/kg on day 1) plus miltefosine (2.5 mg/kg on days 2–8) in India achieved six months cure in 98% (93–99%) of patients aged 6–58 years [7]. At the time of this trial’s design, single and multiple doses of AmBisome were under evaluation in eastern Africa. The recommended regimen of AmBisome monotherapy in Asia is 3-5mg/kg over 3–5 days (total dose of 15mg/kg) or 10mg/kg as single dose administration, whereas in eastern Africa a total dose of 30mg/kg is needed to achieve satisfactory efficacy. On the other hand, to make this combination treatment more field-adapted, one single administration of AmBisome was proposed. A high dose and longer duration of AmBisome and miltefosine respectively were needed in the region, however 10mg/kg AmBisome was considered to be the maximum dose to be used as single administration due to safety concerns. Repeated doses of AmBisome and longer duration of miltefosine would diminish treatment practicality, potentially impact compliance and increase the cost of the regimen. AmBisome 10mg/kg was therefore considered the appropriate option to be administered as a single dose in eastern Africa, in combination with either miltefosine or SSG.

Miltefosine administered as a 28 day course of 2.5mg/kg/day achieved 94% (95% CI: 91–97%) cure at six months post end of treatment in patients 12 years and above in India [9]. In a subsequent study in Indian children aged 2–11 years, cure at six months was 94% (86–98%) [10]. In HIV negative adult males (15 years and above) in Ethiopia, this regimen achieved lower cure at six months in 89% (81–94%) of patients [11]. Most detailed pharmacokinetic data for miltefosine came from a relatively healthy adult European patient group with cutaneous leishmaniasis [12]. While little is known about miltefosine pharmacokinetics in adult or paediatric patients in general, no such data are available in African VL patients. Regional pharmacokinetic data are needed to guide further dose optimization of miltefosine (combination) therapy for VL in eastern Africa. SSG 10-day regimen was defined based on its well characterized efficacy in the region [4], to reduce hospitalization and improve the safety profile of the treatment.

We report the results of a phase II randomized trial, conducted in Africa, to assess the safety, efficacy and pharmacokinetic properties of two 11-day treatment regimens combining AmBisome with SSG and AmBisome with miltefosine, and a 28-day miltefosine monotherapy [13]. The hope was that at least one regimen would reach 90% efficacy at initial and definitive cure.

## Methods

### Trial design

The trial used a sequential design with a triangular continuation region [14]. The null hypothesis was that the proportion cured at day 28 (*p*) is less than or equal to a value *p*_0_ which we set to 75%. The alternative hypothesis is that *p*>*p*_0_. If the upper boundary is crossed during an interim analysis, then the null hypothesis is rejected and we conclude *p*>75%. Crossing the lower boundary at the time of an interim analysis implies that null hypothesis (proportion cured ≤75%) is not rejected and there is specified power to exclude a proportion cured of *p*_a_, for which we chose a value of 90%. The type I error rate and power of the study were pre-specified as 5% and 95%, respectively (α = β = 0.05). Interim analyses were specified after every 15 patients in each arm. The maximum sample size per arm was 63.

### Participants

Patients were recruited from Kimalel Health Centre in Kenya (Baringo district), and Dooka and Kassab hospital (Gedaref State) in Sudan. These study sites are located in areas of stable endemicity. Eligible patients were HIV negative, and aged between 7 and 60 years with parasitologically confirmed VL who signed an informed consent (if aged 18y and over) or whom the parent or legal guardian consented to participate in the study (if under 18y). The target population was primary cases, so known relapse cases, or receipt of any anti-leishmanial drugs in the previous 6 months, was an exclusion criterion. Other exclusion criteria were: severe protein and/or caloric malnutrition defined as kwashiorkor or marasmus in children and BMI <15 in adults; previous history of hypersensitivity reaction to SSG or amphotericin B; concomitant severe infection such as TB or other serious underlying disease which would preclude evaluation of patients response to the study medication; other conditions associated with splenomegaly such as schistosomiasis; previous history of cardiac arrhythmia or an abnormal ECG; Hb<5 g/dL; WBC <10^3^/mm^3^; platelets <40,000/mm^3^, abnormal liver function tests (ALT and AST) of more than three times the upper limit of the normal range, serum creatinine outside the normal range for age and gender, and major surgical intervention within two weeks prior to enrolment. Due to the potential teratogenicity of miltefosine, females of child bearing age were also excluded.

### Interventions

The three treatment regimens were as follows:

- AmBisome 10 mg/kg single dose (IV) on day 1 followed by 10 days of SSG (IM) 20 mg/kg from day 2–11.
- AmBisome 10 mg/kg single dose (IV) on day 1 followed by 10 days of miltefosine 2.5mg/kg/day (oral) from day 2–11 (up to a maximum dose of 150mg)
- Miltefosine 2.5 mg/kg/day (oral) from day 1–28 (up to a maximum dose of 150mg).

AmBisome (liposomal amphotericin B, 50 lyophilized powder in vials, Gilead Pharmaceuticals, USA) was given as a single dose on day 1 at a dose of 10 mg/kg body weight, infused in 5% dextrose over 1–2 hours. Miltefosine (Impavido, Zentaris) was provided as foil-wrapped blister packs. The dose was calculated on a basis of 2.5 mg/kg body weight daily, up to a maximum of 150 mg. However, since 10 and 50 mg capsules were available, the actual doses given were: 30mg for 10-<14kg; 40mg for 14-<18kg; 50mg for 18-<22kg; 60mg for 22-<26kg; 70mg for 26-<30kg; 100mg for 30-<50kg and 150mg for ≥ 50kg. This resulted in a dose range of 2.0–3.33 mg/kg/day of miltefosine. SSG (30 ml vials, each containing 100 mg/ml SSG, produced by Albert David, India) was given as an intramuscular (IM) injection once daily, in a dose of 20 mg/kg body weight.

Rescue treatment was AmBisome 30 mg/kg Intravenously (IV) split into multiple doses (according to country protocol) or SSG 20 mg/kg IM for 30–60+ days for patients not responding to initial rescue treatment or for patients requiring treatment for severe PKDL.

Patients who did not meet inclusion criteria were offered free treatment outside the trial, according to national treatment protocols [15]. All patients were offered counseling and screening for HIV. Patients who tested positive were referred for appropriate treatment to the national HIV control programme and treated according to national treatment guidelines, surveillance and follow up according to the national protocol for HIV positive patients.

### Outcomes

Two efficacy endpoints were defined. The primary endpoint was parasitological cure at Day 28 (initial cure), determined as absence of parasites on microscopy. This was used for interim analysis decisions. Patients who died or required rescue before study treatment could be completed were considered initial treatment failures. The secondary endpoint was assessed at Day 210 (six months post end of treatment), representing final (or definitive) cure status. This was defined as lack of VL signs and symptoms, and no requirement for rescue treatment during the trial. Any patients with signs or symptoms of VL at any time during participation in the trial underwent confirmatory parasitological testing.

Slow responders were defined as patients who had not cleared parasites at Day 28 (D28), but who were clinically well, did not require rescue treatment at D28 and remained clinically well throughout follow-up. These patients had a subsequent parasitological assessment at D56. If microscopy was positive, the patient received rescue medication regardless of clinical presentation. The treatment outcome was classified as failure from the time point rescue was received.

Parasitological assessment by microscopy was done on lymph node aspirates (Dooka, Kassab), spleen aspirates (Kimalel) or bone marrow samples (all sites). In Kimalel, 49 patients who had unpalpable spleen on D28 had bone marrow aspirate while 27 patients had splenic aspirate. Aspirates were smeared on two slides per sample, stained and graded according to the standard logarithmic criteria.

Safety outcomes were the number (%) of patient experiencing a serious adverse event at any time, frequency of adverse event within 60 days of treatment onset and an adverse drug reaction (ADR) within 60 days.

### Sample size

The study was designed and analyzed according to sequential methods, which have been developed to allow for discrete data analysis after a pre-specified number of patients are recruited. The triangular test is one such method and uses straight line stopping boundaries [13]. The continuation region is closed, which ensures a maximum sample size.

A minimum sample size of 30 per arm was imposed to allow for adequate PK assessment. The trial was non-comparative and the sequential analysis was applied to each arm independently, allowing them to potentially stop at different times.

### Randomization

Subjects were randomly allocated using block randomization, stratified by site (Dooka, Kassab and Kimalel). Site investigators were blinded to block size and codes were concealed in sealed sequentially numbered, opaque envelopes under the control of the site investigator. The treating physician and patients were aware of the treatment given; miltefosine is oral medication and AmBisome and SSG are administered intravenously (IV) and IV or intramuscularly (IM) respectively. The laboratory technologists reading the slides were blind to treatment allocation.

### Pharmacokinetics & pharmacodynamics

The pharmacokinetics of miltefosine were assessed in the two arms receiving miltefosine. In the AmBisome + miltefosine arm human sodium heparin samples were nominally collected on day 2, 4, 7, 11, 60, 210 (adults) or day 2, 7, 11, 60, 210 (children); and in the miltefosine monotherapy arm on day 1, 3, 7, 14, 21, 28, 60, 210 (adults) or day 1, 7, 14, 28, 60, 210 (children). On the first day of miltefosine treatment, blood samples were drawn prior to the first dose, 4 hours and 8 hours post first dose; whereas for all other time points blood samples were drawn prior to the first miltefosine administration of that day. Samples were stored and transported frozen at minimally -20°C until analysis. Sample preparation and miltefosine quantification were performed using a validated liquid chromatography tandem mass spectrometry assay with a lower limit of quantitation of 4 ng/mL (LC-MS/MS)[16]. End-of-treatment concentrations of miltefosine were compared with a Welch two sample *t*-test.

Repeated measurements of the *Leishmania* parasite load in whole blood were performed using a qRT-PCR method targeting *Leishmania* kDNA. These pharmacodynamic samples were collected from all participants in all three treatment arms prior to treatment and on Day 3, 7, 14, 28, 60, 210. DNA/RNA isolation was performed partially on site using a modified Boom method [17], where silica samples were stored and transported at minimally -20°C until the moment of further extraction and analysis. The qRT-PCR analysis was performed using a Bio-Rad CFX-96 real-time machine (Bio-Rad, Veenendaal, the Netherlands). Parasite clearance rates were calculated as relative decreases from baseline of all patients who had a detectable parasite load at baseline. A linear mixed-effects model was fitted in R using the maximum likelihood method, with treatment day and arm as fixed effects and subject as a random effect.

### Statistical analysis

Data analysis was performed using STATA, version 13 [18]. The primary analysis was by intention-to-treat (ITT).

Interim analyses were conducted once every 15 patients had reached D28 primary endpoint assessment, using all available data and were based on the ITT analysis population. During each interim analysis, triangular region tests were performed [14] and a decision was taken whether to continue or stop recruitment in each arm based on the position of the test statistic being within or outside the triangular region.

Once a decision was reached for each arm to stop, a point and interval estimate for the overall proportion cured at Day 28 (*p*) was obtained following Whitehead [19] based on all patient data using the ITT analysis population.

The proportion of patients cured at day 210 is subject to sequential stopping as far as day 28, but not thereafter. To take account of this, the probability of treatment success at D210 was estimated by a probability tree argument, using the delta method for its standard error [20]. This method takes account of the occurrence of slow response to treatment and relapse captured between D28 and D210. Relapse patients are those without detectable parasites at D28, but who develop signs and symptoms of VL during the follow-up period and have a confirmatory parasitological diagnosis anytime between D28 and D210. This analysis was based on the ITT analysis population.

A pre-specified subgroup analysis was to compare cure by sites using a χ^2^ or Fisher’s exact test at D210, based on the ITT analysis population. Due to the relative geographical proximity and small numbers, data from the Sudanese sites were combined, post hoc, to conduct the subgroup analysis by country. Also, efficacy at Day 210 is compared by age groups split at 12 years, rather than the pre-specified 18 years.

All adverse events were coded to have lower level preferred term, higher level term and system organ class classifications according to MedDRA, Version 12.0.

Safety outcomes were calculated as risk measures (number and percent of patients out of those randomized) where patients with multiple AEs or ADRs were only counted once. The incidence of ADRs was calculated as the number and percentage of patients experiencing each type of AE.

### Ethical approval

Ethical approval was obtained from national and local Ethics Committees in Kenya and Sudan prior to the start of the trial in each country. Ethical approval was also granted by LSHTM’s Ethics Committee (#5543), and the Academic Medical Center Medical Ethics Committee issued a 'declaration of no objection'. Study participants or their parents/guardians (for children) gave a written signed informed consent before enrollment into the study.

The study was registered with ClinicalTrials.gov, number NCT01067443.

## Results

### Participants

**Fig 1:**
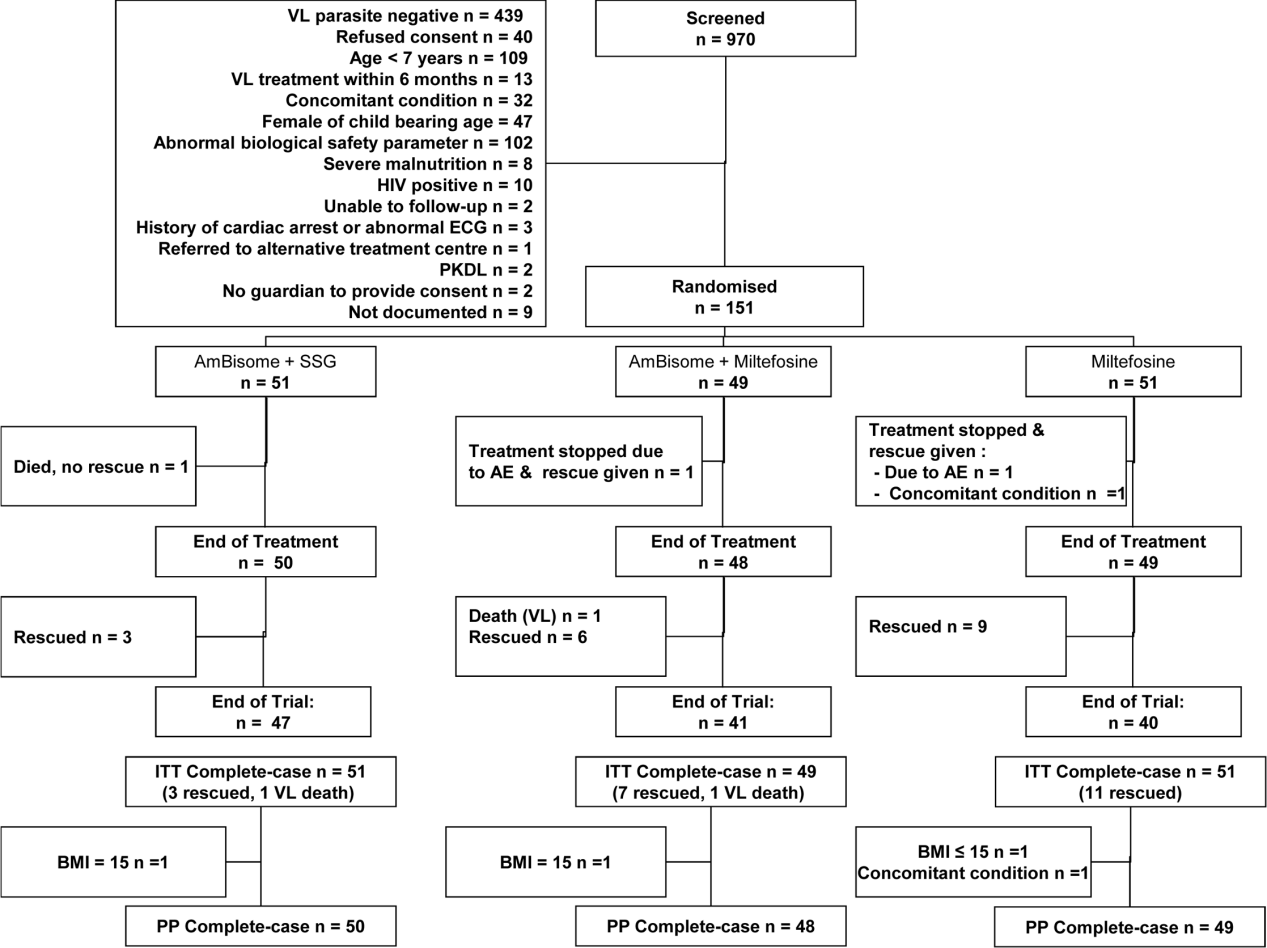
Participant Flow.

A total of 151 patients were enrolled in the study (Fig 1). The most common reasons for exclusion amongst patients with detectable parasites (n = 531) were age less than seven years (20.5%), abnormal biological parameters (19.2%), being female of child bearing age (8.9%) and refusal of consent (7.5%). Patients were recruited from May 2010 to Feb 2012. All patients remained hospitalized from screening until completion of D28 assessment. Follow up was completed in Oct 2012. The three arms appeared balanced with respect to baseline characteristics (Tables 1–4).

**Table 1:** Baseline demographic characteristics.

		AmBisome + SSG	AmBisome + miltefosine	Miltefosine
		N = 51	N = 49	N = 51
Age (years) n (%)	mean (SD)	15 (8)	14 (6)	15 (8)
	7–17	35 (69)	35 (71)	37 (73)
	18–60	16 (31)	14 (29)	14 (27)
Sex n (%)	Female	14 (27)	9 (18)	5 (10)
	Male	37 (73)	40 (82)	46 (90)
Site n (%)	Dooka	19 (37)	18 (37)	20 (39)
	Kassab	5 (10)	6 (12)	7 (14)
	Kimalel	27 (53)	25 (51)	24 (47)

**Table 2:** Baseline biological markers.

		AmBisome + SSG	AmBisome + miltefosine	Miltefosine
		N = 51	N = 49	N = 51
Weight	mean (SD)	35.2 (14.5)	34.3 (13.4)	36.7 (13.4)
(kg)	median (range)	31 (15–69)	30 (15–59)	31 (16–65)
Temperature	mean (SD)	37.5 (1.1)	37.5 (1.1)	37.6 (1.3)
(°C)	median (range)	37.3 (35.7–40)	37.5 (36–40.1)	37.6 (35.8–40.2)
Heart Rate	mean (SD)	100.9 (10.6)	103 (13.7)	99.1 (11.4)
(beats/min)	median (range)	100 (80–130)	100 (68–130)	100 (80–128)
Spleen Size	mean (SD)	8.5 (4.4)	8.4 (5.6)	8.2 (3.9)
(cm)	median (range)	8 (0–18)	7 (0–22)	8 (0–18)
Liver Size***	mean (SD)	2.3 (2.4)	2.5 (2.3)	2.7 (2.4)
(cm)	median (range)	2 (0–10)	2 (0–8)	2 (0–10)
Systolic BP	mean (SD)	100.9 (10.4)	95.7 (9.5)	99.4 (7.7)
(mm Hg)	median (range)	100 (80–130)	90 (80–120)	100 (85–120)
Diastolic BP	mean (SD)	62.2 (7)	61.2 (7.1)	63 (7.3)
(mm Hg)	median (range)	60 (50–80)	60 (50–80)	60 (50–80)
Nutritional Status n (%)	Severely underweight (BMI*< 15)	5 (10)**	9 (18)**	8 (16)**
	Underweight (BMI 15–18.4)	23 (45)	18 (37)	16 (31)
	Normal (BMI 18.5–24.9)	22 (43)	22 (45)	27 (53)
	Obese/overweight (BMI > 24.9)	1 (2)	0 (0)	0 (0)

*BMI in kg/m^2^

**nutritional status categorization derived by post-hoc determination of WHO standardized value, as opposed to the BMI threshold in the severely underweight exclusion criterion.

*** Liver size was measured below right costal margin

**Table 3:** Baseline parasite count.

	AmBisome + SSG	AmBisome + miltefosine	Miltefosine
	N = 51	N = 49	N = 51
Parasite Count Oil Immersion x100: n (%)			
> 100,000/1000 (6+)	2 (4)	4 (8)	6 (12)
10,001–100,000/1000 (5+)	12 (24)	8 (16)	10 (20)
1,001–10,000/1000 (4+)	9 (18)	14 (29)	7 (14)
101–1,000/1000 (3+)	4 (8)	3 (6)	5 (10)
11-100/1000 (2+)	2 (4)	2 (4)	5 (10)
1-10/1000 (1+)	22 (43)	18 (37)	18 (35)
0	0 (0)	0 (0)	0 (0)

**Table 4:** Baseline laboratory parameters.

		AmBisome + SSG	AmBisome + miltefosine	Miltefosine
		N = 51	N = 49	N = 51
Haemoglobin	mean (SD)	7.4 (1.8)	7 (1.3)	7 (1.3)
(g/dL)	median (range)	7.3 (5–12.9)	6.9 (5–9.7)	6.8 (5–10.3)
White-cell Count	mean (SD)	2.7 (1.3)	2.3 (1.2)	2.6 (1)
(x10^3^/μL)	median (range)	2.6 (1–7.4)	2.1 (1–5.5)	2.4 (1–5.2)
Platelets	mean (SD)	130 (90)	101 (54)	114 (65)
(x10^3^/μL)	median (range)	104 (47–471)	88 (39–288)	100 (35–313)
AST	mean (SD)	48 (25.9)	56.2 (31.7)	49 (29.9)
(U/L)	median (range)	44 (8–112)	50 (3–115)	43.5 (3–117)
ALT	mean (SD)	34.3 (20.5)	40.5 (27.1)	32.9 (20.7)
(U/L)	median (range)	25 (2–87)	32 (4–102)	27.5 (7–102)
Creatinine	mean (SD)	73.7 (26)	71.2 (24.7)	78 (21.2)
(μmol/L)	median (range)	75 (35.4–129)	70.7 (26.5–114.9)	79 (35.4–132)
Blood urea nitrogen	mean (SD)	5.7 (3.2)	5.8 (2.8)	5.7 (3.1)
(mmol/L)	median (range)	5.3 (1.7–12.5)	6.1 (1.3–11.4)	5 (1.3–17.1)
Alkaline phosphatase	mean (SD)	222 (196)	211 (157)	197 (117)
(U/L)	median (range)	184 (10–1272)	169 (62–817)	176 (35–464)
Bilirubin	mean (SD)	8.3 (4.8)	8.3 (4.6)	8.7 (5.2)
(total, mmol/L)	median (range)	6.8 (2–27.4)	6.8 (1.7–22)	6.8 (3–27.4)
Sodium	mean (SD)	137 (7)	135 (8)	134 (7)
(mmol/L)	median (range)	137 (120–159)	136 (110–149)	133 (120–149)
Potassium	mean (SD)	3.8 (0.4)	3.8 (0.4)	3.9 (0.4)
(mmol/L)	median (range)	3.8 (2.9–4.6)	3.8 (2.9–4.6)	3.9 (2.8–4.9)
Magnesium	mean (SD)	0.9 (0.3)	0.9 (0.2)	0.9 (0.2)
(mmol/L)	median (range)	1 (0.2–1.9)	1 (0.2–1.6)	1 (0.4–1.5)

ALT: Alanine transaminase; AST: aspartate aminotransferase

### Analysis populations

Data from all 151 enrolled patients were available for the intention-to-treat analysis: 51 for the AmBisome + SSG arm, 49 for the AmBisome + miltefosine arm and 51 for the miltefosine monotherapy arm. There were four major protocol deviations. In three patients (one from each treatment arm), body mass index at baseline was recorded as 15kg/m^2^. One patient in the miltefosine monotherapy arm was discovered to have epilepsy and should also have been excluded during screening due to severe concomitant condition.

Therefore, 147 patients were included in the per-protocol (PP) population, 50 for the AmBisome + SSG arm, 48 for the AmBisome + miltefosine arm and 49 for the miltefosine monotherapy arm.

### Interim analyses

**Fig 2:**
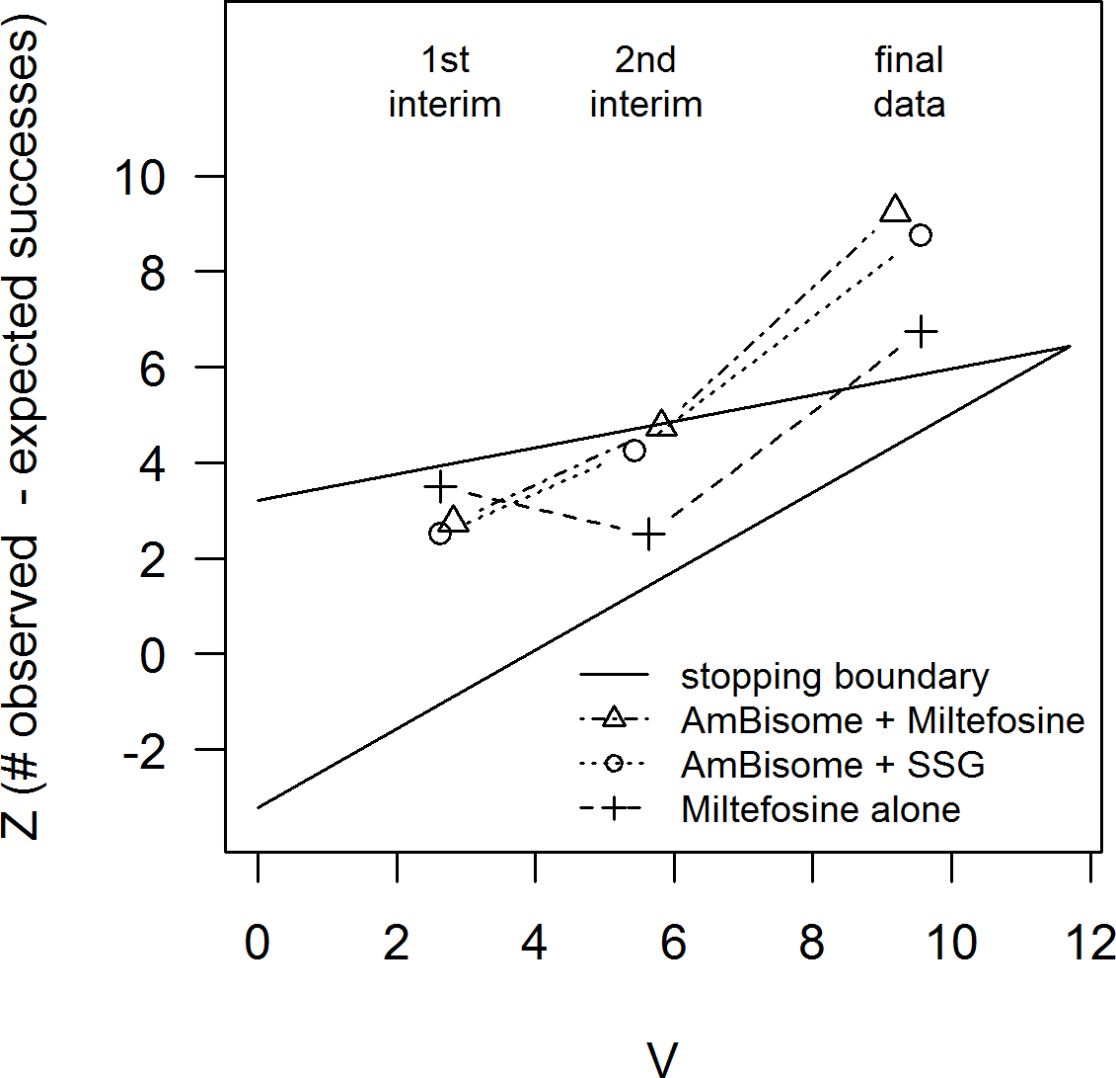
Sequential stopping for the three arms. The horizontal axis (*V*) is proportional to sample size. The vertical axis (*Z*) is the observed minus expected number of cures, so higher values are more favourable. Each arm is shown by a line with three points representing, from left to right, the first interim analysis (decision: continue for all arms), the second interim analysis (decision: continue for all arms) and the final analysis. The final analysis (shown here) includes patients whose follow-up was still in progress at the time of the third interim analysis, and confirmed the ‘stop’ decisions. Having all stopped at the same analysis, the point estimates of proportion cured are the same for all arms (85%), as are the 95% confidence intervals (77–97%). Based on the probability tree method, the point estimate at day 210 for AmBisome + SSG is 87% (95% CI 77–97%); for AmBisome + Miltefosine it is 77% (64–90%) and for Miltefosine 72% (60–85%).

The first and second interim analyses indicated that all arms should continue (Fig 2; Table 5). The decision to stop recruitment was made after the third sequential analysis for promising D28 efficacy in all three arms based on crossing the upper triangular boundary.

**Table 5:** Outcomes and events during treatment: Efficacy.

	AmBisome + SSG			AmBisome + Miltefosine			Miltefosine		
**Interim analysis (D28: ITT)**	**N**	**Cured, n**	**Decision**	**N**	**Cured, n**	**Decision**	**N**	**Cured, n**	**Decision**
1	14	13	Continue	15	14	Continue	14	14	Continue
2	29	26	Continue	31	28	Continue	30	25	Continue
3	44	41	Stop	44	41	Stop	47	42	Stop
**Cumulative analysis (D28)**	**N**	**Cured, n**	% (95% CI*)	**N**	**Cured, n**	% (95% CI*)	**N**	**Cured, n**	% (95% CI*)
ITT	51	47	85% (73–92)	49	46	85% (73–92)	51	45	85% (73–92)
**Cumulative analysis (D210)**	**N**	**Cured, n**	% (95% CI*)	**N**	**Cured, n**	% (95% CI*)	**N**	**Cured, n**	% (95% CI*)
ITT	51	47	87% (77–97)	49	40	77% (64–90)	51	38	72% (60–85)

* Efficacy and corresponding 95% CI estimated taking into account sequential design

### Initial response to treatment (D28 efficacy)

When accounting for the sequential trial design, cumulative efficacy at D28 was 85% (95% CI: 73%–92%) in all three arms (see Table 5). Results from the per protocol analysis were similar to the ITT population.

### Final cure at 6 months post end of treatment (D210 efficacy)

No patients were lost to follow up. One slow responder occurred in each treatment arm. Relapse occurred in one, seven and eight patients in the AmBisome + SSG, AmBisome + Miltefosine and Miltefosine arms respectively. When accounting for the sequential trial design and change of status between D28 and D210, the D210 efficacy was 87% (95% CI: 77–97%) for the AmBisome + SSG arm, 77% (95% CI: 64–90%) for the AmBisome + miltefosine arm and 72% (95% CI: 60–85%) for the miltefosine arm (see Table 5).

### Sub-group analysis

D210 cure was consistently lower in Sudan (Table 6) than in Kenya for each treatment, especially for the AmBisome + miltefosine arm (p = 0.074). The age distribution differed by country with more younger patients recruited in Sudan; 49 (65%) Sudanese patients were aged less than 12 years compared to 25 (33%) Kenyan patients (chi-squared test p<0.0001). Age, as a possible proxy for weight, could therefore be a realistic biological explanation for a difference in treatment response by country, although additional differences in parasite susceptibility and host factors cannot be excluded. In the present study population, age <12y was correlated with weight <30kg. Therefore post-hoc stratified analyses were conducted by age group (<12 years, ≥12 years) and country. An age cut-off of 12 years was chosen based on the observed differences in drug exposure between those two groups. Cure rates were consistently lower in patients aged less than 12 years in each arm in both countries (Table 6).

**Table 6:** Stratified D210 efficacy.

		AmBisome + SSG	AmBisome + Miltefosine	Miltefosine
Country	Sudan	21/24 (88%)	17/24 (71%)	18/27 (67%)
	Kenya	26/27 (96%)	23/25 (92%)	20/24 (83%)
Fisher’s exact test p-value (2-sided)		0.331	0.074	0.211
Age	Less than 12 years	22/25 (88%)	20/27 (74%)	13/22 (59%)
	12 years and above	25/26 (96%)	20/22 (90%)	25/29 (86%)
Fisher’s exact test p-value (2-sided)		0.350	0.159	0.050
Sudan	Less than 12 years	12/15 (80%)	13/19 (68%)	9/15 (60%)
	12 years and above	9/9 (100%)	4/5 (80%)	9/12 (75%)
Kenya	Less than 12 years	10/10 (100%)	7/8 (88%)	4/7 (57%)
	12 years and above	16/17 (94%)	16/17 (94%)	16/17 (94%)

Data are number cured / number randomised (%). As estimates of cure rates, these percentages do not take into account the sequential design.

### Safety

There were four SAEs, occurring in two patients in each of the AmBisome containing arms (4% of patients randomised to each short-course arm; Table 7). Three patients discontinued treatment.

**Table 7:** Safety summary.

	AmBisome + SSG	AmBisome + Miltefosine	Miltefosine
**Number of patients**	**51**	**49**	**51**
*SAE*, *n (%)*	2 (4)	2 (4)	0 (0)
SAE related to study drug, *n (%)*	1 (2)	1 (2)	0 (0)
Deaths, n (%)	1 (2)	1 (2)	0 (0)
TEAE, *n (%)*	41 (80)	44 (90)	46 (90)
TEADR, *n (%)*	37 (73)	38 (78)	40 (78)
Treatment stopped due to AE	0 (0)	2* (4)	1 (2)
AE during AmBisome infusion	11 (22)	9 (18)	-
Vomited any scheduled dose, n (%)	-	10 (20)	11 (22)
Repeatedly vomited the same scheduled dose	-	1 (2)	2 (4)
Vomited more than one scheduled dose	-	1 (2)	5 (10)

*Only one of these received rescue and is shown as such in the flowchart.

TEAE: Treatment Emergent Adverse Event; TEADR: Treatment Emergent Adverse Drug Reaction

One SAE in each AmBisome arm was a serious adverse drug reaction (SADR); in the AmBisome + SSG arm, severe anaemia resulted in death at day 20, and in the AmBisome + miltefosine arm, renal failure at day 3 which was resolved. The two unrelated SAE were upper respiratory tract infection and pneumonia. There were two deaths, one in each combination arm. These were severe pneumonia in the AmBisome + Miltefosine arm which was considered not related to study drug and severe anemia in the AmBisome + SSG arm which was considered possibly related.

The proportion of patients with at least one treatment emergent adverse event (TEAE) was between 80 and 90% in each arm. The proportion of patients with at least one ADRs was between 73 and 78% in each arm, while the proportion of patients with at least one AE not related to study drug was between 33 and 45%.

Treatment was stopped in 1 patient due to a non-serious, moderate increase in serum creatinine and elevation of blood urea after 14 days of miltefosine monotherapy treatment. Two patients in the AmBisome + miltefosine arm stopped treatment early (due to serious renal insufficiency after 3 days of treatment and moderate non-serious increased blood creatinine after 7 days).

The median number of adverse effects experienced was two per patient for each arm. Of all non-serious drug related events, three were severe and occurred in the miltefosine monotherapy arm: two cases of anaemia and one case of gastro-intestinal pain. In the AmBisome + SSG and in the AmBisome + miltefosine arms, all non-serious drug-related events were categorized as mild to moderate.

Approximately 20% of patients in each arm treated with AmBisome experienced adverse events during infusion, and approximately 20% of patients in each arm treated with miltefosine vomited at least one dose. The overall occurrence of vomiting was 21% in all patients treated with miltefosine. The occurrence of repeated vomiting of the same dose was relatively low (6% of patients overall in both miltefosine arms). All treatment arms showed CTCAE grade 1 or grade 2 aspartate aminotransferase increase (14–29%) and grade 1 hypomagnesaemia (14–20%) (Table 8).

**Table 8:** Incidence of adverse drug reactions.*

	AmBisome + SSG			AmBisome + Miltefosine			Miltefosine		
	<12 years	≥12 years	Total	<12 years	≥12 years	Total	<12 years	≥12 years	Total
	23	28	51	24	25	49	20	31	51
**BLOOD AND LYMPHATIC SYSTEM DISORDERS**									
ANAEMIA	2 (9%)	1 (4%)	3 (6%)	4 (17%)	0 (0%)	4 (8%)	5 (25%)	1 (3%)	6 (12%)
**GASTROINTESTINAL DISORDERS**									
VOMITING	0 (0%)	1 (4%)	1 (2%)	8 (33%)	3 (12%)	11 (22%)	6 (30%)	7 (23%)	13 (25%)
**GENERAL DISORDERS AND ADMINISTRATION**									
PYREXIA	8 (35%)	3 (11%)	11 (22%)	3 (13%)	2 (8%)	5 (10%)	3 (15%)	1 (3%)	4 (8%)
**INVESTIGATIONS**									
ALANINE AMINOTRANSFERASE INCREASED	3 (13%)	2 (7%)	5 (10%)	1 (4%)	2 (8%)	3 (6%)	4 (20%)	5 (16%)	9 (18%)
ASPARTATE AMINOTRANSFERASE INCREASED	6 (26%)	5 (18%)	11 (22%)	5 (21%)	2 (8%)	7 (14%)	6 (30%)	9 (29%)	15 (29%)
BLOOD ALKALINE PHOSPHATASE INCREASED	4 (17%)	3 (11%)	7 (14%)	4 (17%)	0 (0%)	4 (8%)	3 (15%)	2 (6%)	5 (10%)
BLOOD CREATININE INCREASED	1 (4%)	4 (14%)	5 (10%)	1 (4%)	3 (12%)	4 (8%)	1 (5%)	2 (6%)	3 (6%)
BLOOD UREA INCREASED	4 (17%)	1 (4%)	5 (10%)	3 (13%)	3 (12%)	6 (12%)	1 (5%)	1 (3%)	2 (4%)
**METABOLISM AND NUTRITION DISORDERS**									
HYPOKALAEMIA	1 (4%)	5 (18%)	6 (12%)	2 (8%)	3 (12%)	5 (10%)	0 (0%)	0 (0%)	0 (0%)
HYPOMAGNESAEMIA	4 (17%)	3 (11%)	7 (14%)	7 (29%)	0 (0%)	7 (14%)	5 (25%)	5 (16%)	10 (20%)
HYPONATRAEMIA	2 (9%)	1 (4%)	3 (6%)	3 (13%)	2 (8%)	5 (10%)	3 (15%)	0 (0%)	3 (6%)
**RESPIRATORY, THORACIC AND MEDIASTINAL DISORDERS**									
EPISTAXIS	5 (22%)	0 (0%)	5 (10%)	1 (4%)	1 (4%)	2 (4%)	2 (10%)	2 (6%)	4 (8%)
**CARDIAC DISORDERS**									
ARRYTHMIA SUPRAVENTRICULAR	0 (0%)	0 (0%)	0 (0%)	0 (0%)	1 (4%)	1 (2%)	0 (0%)	0 (0%)	0 (0%)
SINUS ARRYTHMIA	1 (4%)	1 (4%)	2 (4%)	3 (13%)	0 (0%)	3 (6%)	0 (0%)	0 (0%)	0 (0%)
SINUS BRADYCARDIA	0 (0%)	1 (4%)	1 (2%)	0 (0%)	0 (0%)	0 (0%)	0 (0%)	0 (0%)	0 (0%)
**INVESTIGATIONS**									
ELECTROCARDIOGRAM ABNORMAL	0 (0%)	0 (0%)	0 (0%)	1 (4%)	0 (0%)	1 (2%)	0 (0%)	0 (0%)	0 (0%)
**RENAL AND URINARY DISORDERS**									
RENAL IMPAIRMENT	0 (0%)	0 (0%)	0 (0%)	1 (4%)	0 (0%)	1 (2%)	0 (0%)	0 (0%)	0 (0%)
RENAL FAILURE	0 (0%)	0 (0%)	0 (0%)	0 (0%)	1 (4%)	1 (2%)	0 (0%)	0 (0%)	0 (0%)

* All events which occurred with more than 5% overall frequency, together with all cardiac disorders, and renal disorders.

Note: Causality assessment was based on investigator judgment. According to protocol all events that were not recorded as ‘Not Related’ were considered ‘Adverse Drug Reactions’

The only arm to contain SSG (combined with AmBisome) showed low levels of cardiac disorders (<5%), similar to that of the AmBisome + MF arm. PKDL occurred in eight Sudanese patients, 2 in the AmBisome+SSG arm, 1 in the AmBisome+Miltefosine arm and 5 in the miltefosine monotherapy arm. All cases were mild or moderate and did not require treatment.

### Pharmacokinetics & pharmacodynamics

**Fig 3:**
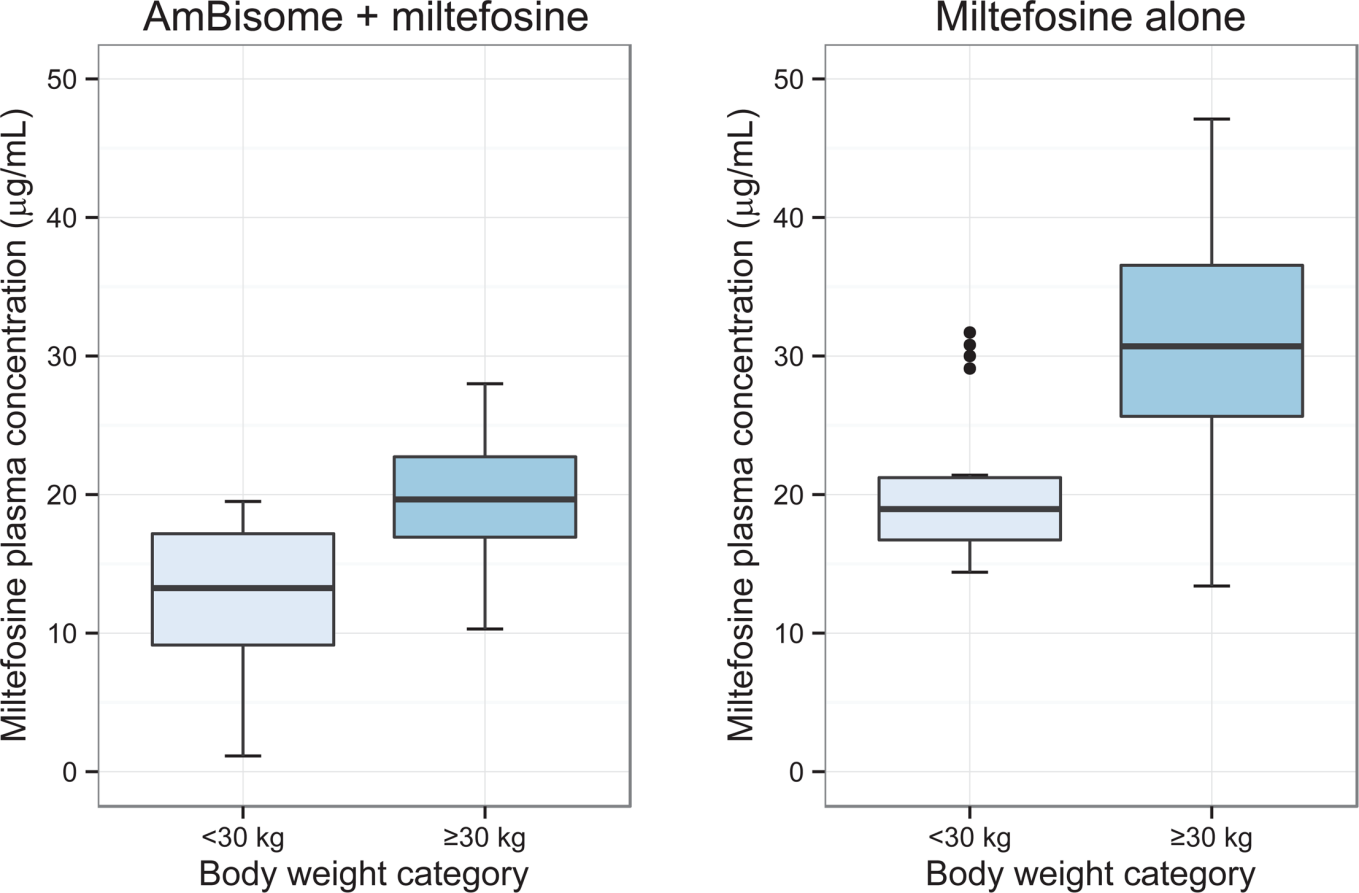
Comparison of the end-of-treatment miltefosine plasma concentrations between body weight categories. The left plot shows the AmBisome + miltefosine arm (10 days of miltefosine), the right plot the miltefosine alone arm (28 days of miltefosine).

Miltefosine end of treatment concentrations were available for 42 patients from the AmBisome + miltefosine arm and for 45 patients from the miltefosine alone arm. Miltefosine keeps accumulating until the end of treatment, so the end-of-treatment concentration (day 11 for the AmBisome + miltefosine arm and day 28 for the miltefosine alone arm) is representative for total exposure. The mean (SD) miltefosine concentration at the end-of-treatment was 16.47 (5.89) μg/mL for the AmBisome + miltefosine arm and 26.42 (8.66) μg/mL for the miltefosine alone-arm. There were no significant differences in miltefosine end-of-treatment concentrations between Sudanese and Kenyan patients. As depicted in Fig 3, patients with a body weight <30 kg (n = 38, of which 97% aged ≤12 yrs) were significantly less exposed to miltefosine than patients with a higher body weight of ≥30 kg (n = 49, of which 84% aged >12 yrs): for the AmBisome + miltefosine arm the difference in end-of-treatment concentration was 36% (p<0.0001) and for the miltefosine alone arm 32% (p<0.0001).

**Fig 4:**
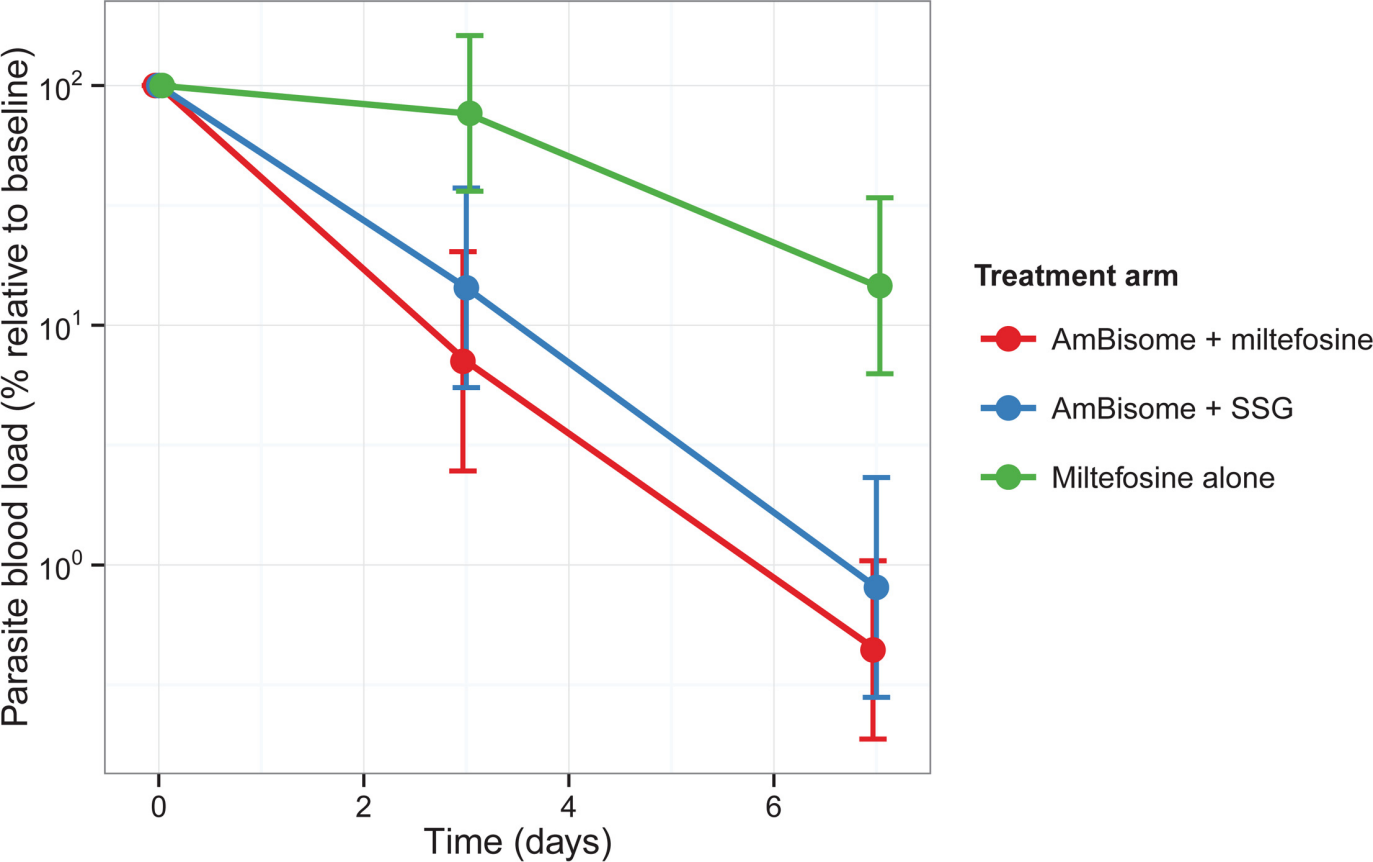
Parasite clearance from the blood in the first week of treatment. All parasite blood loads are relative to the individual parasite load at baseline. The full lines indicate the mean and the error bars its 95% confidence interval, stratified per treatment arm.

In total, for 77% of all patients there was a blood parasite load available at baseline, with various reasons for non-availability (e.g. no baseline sample available, issues with DNA extraction or no parasite load detectable). Only the first week of treatment was taken into account to assess the parasite clearance rate, since levels dropped to undetectable levels >1 week of treatment for many patients. The effect of the treatment arm on the parasite clearance rate in the first week of treatment was evaluated and is illustrated in Fig 4. The parasite clearance rate was slower in the miltefosine alone arm compared to the AmBisome + miltefosine arm (p<0.0001), while there was no significant difference between the two combination arms (p = 0.605). After 1 week of treatment, almost all individuals in both combination treatment arms had regressed towards 0% parasite load, while the miltefosine monotherapy arm was on average still >10% of the initial parasite load level (Fig 4).

A more extensive in-depth analysis of the pharmacokinetic and pharmacodynamics results of this study will be reported elsewhere.

## Discussion

This was the first phase II randomized trial of short course combination therapies containing AmBisome for VL in Africa. Neither of the two AmBisome combinations (single daily dose of AmBisome with 10 days of SSG or miltefosine) nor the 28 day miltefosine monotherapy (previously used in India) achieved more than 90% cure. There were no unexpected safety signals detected in the trial, although there is very little power to detect unexpected events given the small number of patients treated. Our recent study showed that multiple daily doses of 3 mg/kg body weight of AmBisome maybe more beneficial to parasite clearance than a single 10 mg/kg dose at day 1 [8], suggesting that a more frequent administration of AmBisome may result in higher cure rates.

Pharmacodynamic data showed faster parasitic clearance with both AmBisome combinations than with miltefosine monotherapy. Our study was not designed or powered to detect differences in efficacy between adults and children, however patients with low body weight (<30kg), almost all of whom were children under 12 years of age, were found to be significantly underexposed to miltefosine compared to those with higher body weight, whether treated with miltefosine alone or in combination with AmBisome.

The lack of efficacy compared to that seen previously in Southeast Asia, particularly with regard to miltefosine, could be due to genetic diversity and differences in drug susceptibility between east African and Indian *Leishmania* strains. However, results from Nepal and India recently demonstrated a high relapse rate 6–12 months after miltefosine monotherapy (75–90% efficacy), and more frequently in patients under the age of 15 years, suggesting a possible drug underexposure effect [21,22]. Pharmacokinetic studies on miltefosine exposure in adults and children from both countries show that the current 2.5 mg/kg/day miltefosine monotherapy dose results in low exposure in children and that a proposed allometric dosing algorithm could provide a higher and more optimal exposure in both adults and children [23]. Similar lower drug exposure in children has been observed for antimonials (meglumine antmoniate) in cutaneous leishmaniasis using a linear weight-adjusted dosage[24].

Our results confirmed a good tolerability of all treatments tested and no major safety concerns were identified. Despite the higher exposure of miltefosine in adults, this does not seem to be associated with higher safety risks. The frequency and severity of increase in creatinine observed in this trial were not different from what had been already described.

In conclusion, as none of the treatment regimens tested achieved target efficacy they will not be developed further. Miltefosine, as an oral drug treatment, is still of interest and so an allometric dosing study is now underway to assess the safety and appropriate treatment dose of miltefosine, particularly in children, in eastern Africa (NCT02431143).

This study highlights, once again, the difficulties faced when developing treatments for VL in eastern Africa. In addition to improvements based on existing drugs, early drug discovery efforts are ongoing, and several new chemical entities have been identified which will hopefully give rise to safe, effective, oral treatments for the region in the future.

## Supporting Information

S1 Checklist CONSORT 2010 checklist. Information to be included according to the 2010 Consolidated Standards of Reporting Trials (CONSORT).
(DOC)

S1 Protocol Clinical trial protocol. Protocol for a Phase II Randomized, Parallel Arm, Open-Labeled Clinical Trial to Assess the Safety and Efficacy of the Combination of Sodium Stibogluconate Plus Single Dose Ambisome, Miltefosine Plus Single Dose Ambisome and Miltefosine Alone for the Treatment of Primary Visceral Leishmaniasis in Eastern Africa.
(PDF)
