# Supplementary material for: Efficacy and Safety of AmBisome in Combination with Sodium Stibogluconate or Miltefosine and Miltefosine Monotherapy for African Visceral Leishmaniasis: Phase II Randomized Trial
Source: PLoS Negl Trop Dis. 2016 Sep 14;10(9):e0004880. doi: 10.1371/journal.pntd.0004880 (PMC5023160; doi:10.1371/journal.pntd.0004880)
Supplement: S1 Protocol — Protocol for a Phase II Randomized, Parallel Arm, Open-Labeled Clinical Trial to Assess the Safety and Efficacy of the Combination of Sodium Stibogluconate Plus Single Dose Ambisome, Miltefosine Plus Single Dose Ambisome and Miltefosine Alone for the Treatment of Primary Visceral Leishmaniasis in Eastern Africa. (PDF) [file pntd.0004880.s002.pdf]

DNDi
Drugs for Neglected Diseases *initiative*

CLINICAL TRIAL PROTOCOL

Protocol Title

"A phase II randomized, parallel arm, open-labeled clinical trial to assess the safety and efficacy of the combination of sodium stibogluconate plus single dose AmBisome®, Miltefosine plus single dose AmBisome® and Miltefosine alone for the treatment of primary visceral leishmaniasis in Eastern Africa. "

**Name of product(s)/ Project code**	Sodium Stibogluconate (SSG), Miltefosine, Liposomal Amphotericin B (AmBisome®)
**Drug Class**	*Pentavalent antimonial, alkylphosphocholine, polyene antibiotic*
**Phase**	Phase II
**Indication**	Treatment of primary visceral leishmaniasis (VL) in East Africa
**Protocol Number**	**LEAP 0208**
**Sponsor**	DND*i*, Chemin Louis Dunant, 15, 1202 GENEVA Switzerland<br>Phone: +41 22 906 9230
**Principal Investigator / Coordinating Investigator**	Professor EAG Khalil / Dr AM Musa<br>Institute of Endemic Diseases (IED), University of Khartoum<br><br>Dr Monique Wasunna<br>Kenya Medical Research Institute (KEMRI), Kenya
**Protocol Version / Date**	Final version 26.03.2009
**Protocol Amendment Number / Date**	-

The information contained in this document is confidential. It is to be used by investigators, potential investigators, consultants, or applicable independent ethics committees. It is understood that this information will not be disclosed to others without written authorisation from DNDi, except where required by applicable local laws

List of Investigators

Principal Investigators

Prof Eltahir Khalil	IED, University of Khartoum, Sudan
Dr Ahmed Musa	IED, University of Khartoum, Sudan
Dr Monique Wasunna	KEMRI, Nairobi, Kenya

Co-Investigators

Professor Ahmed EL-Hassan	IED, University of Khartoum, Sudan
Dr Brima Musa Younis	IED, University of Khartoum, Sudan
Dr Abuzaid A Abuzaid	IED, University of Khartoum, Sudan
Dr Mahmoud Mudawi	IED, University of Khartoum, Sudan
Dr Njenga Njoroge	KEMRI, Nairobi, Kenya
Dr Fredrick Kirui	KEMRI, Nairobi, Kenya

Collaborating institutions

Pharmacokinetics

Thomas Dorlo ASK, Amsterdam, Netherlands

Trial statistician

Neal Alexander London School of Hygiene & Tropical Medicine

Project Manager

Dr Manica Balasegaram DND*i* (Sponsor), Geneva, Switzerland

SIGNATURES

DNDi / Sponsor's signature (Medically qualified DNDi Officer)

Signature ________________________ ________________
Date of Signature (DD/mm/YY)

Name	Dr Manica Balasegaram
Title	Project Manager
Institution	Drugs for Neglected Diseases *initiative*
Address	15 Chemin Louis-Dunant, Geneva 1202, Switzerland

Statistician

Signature ________________________ ________________
Date of Signature (DD/mm/YY)

Name	Neal Alexander
Title	Trial Statistician
Institution	London School of Hygiene & Tropical Medicine
Address	Keppel Street WC1E 7HT, United Kingdom

Investigators Signature Page

I have read this protocol and agree that it contains all necessary details for carrying out this trial. I will conduct the trial as outlined herein and will complete the trial within the time designated.
I will provide copies of the protocol and all pertinent information to all individuals responsible to me who assist in the conduct of this trial. I will discuss this material with them to ensure they are fully informed regarding the drug and the conduct of the trial.
I will use only the informed consent form approved by the sponsor or its representative and will fulfill all responsibilities for submitting pertinent information to the Institutional Review Board/Independent Ethics Committee (IRB/IEC) responsible for this trial.
I agree that the sponsor or its representatives shall have access to any source documents from which case report form information may have been generated.

Principal Investigators

Signature ________________________________ ____________________
Date of Signature (DD/mm/YY)

Name Prof. Eltahir Khalil
Title Principal Investigator
Institution Institute of Endemic Diseases
Address University of Khartoum, Sudan

Signature ________________________________ ____________________
Date of Signature (DD/mm/YY)

Name Dr Ahmed Musa
Title Principal Investigator
Institution Institute of Endemic Diseases
Address University of Khartoum, Sudan

Signature ________________________________ ____________________
Date of Signature (DD/mm/YY)

Name Dr Monique Wasunna
Title Principal Investigator/ Coordinating investigator
Institution Kenya Medical Research Institute
Address PO Box 54840, Nairobi, Kenya

TABLE OF CONTENTS

ABBREVIATIONS – GLOSSARY OF TERMS 7
PROTOCOL SYNOPSIS 8
1. BACKGROUND AND STUDY RATIONALE 11
2. STUDY OBJECTIVES AND ENDPOINTS 13
2.1.1. Primary Objective 13
2.1.2. Secondary Objectives 13
2.2. Study Endpoints 14
2.2.1. Primary Endpoint 14
2.2.2. Secondary Endpoint(s) 14
3. STUDY DESIGN AND STUDY DESIGN RATIONALE 14
3.1. Study design 14
3.2. Study duration and duration of subject participation 15
3.3. Rationale of study design 15
4. SELECTION OF SUBJECTS 17
4.1. Inclusion criteria 18
4.2. Exclusion criteria 18
5. SCHEDULE OF EVENTS 20
6. ENROLMENT PROCEDURES 21
7. TREATMENTS 21
7.1. Investigational Product 21
7.2. Doses and treatment regimens 22
7.3. Drugs labelling, packaging 23
7.4. Storage 23
7.5. Blinding and procedures for un-blinding 23
7.6. Concomitant medications 23
8. STUDY ASSESSMENTS 23
8.1. Timing of Assessments 23
8.2. Baseline Assessments 24
8.3. Assessment of Efficacy 24
8.3.1. Assessments performed 25
8.3.2. Laboratory examinations 25
8.4. Other Assessments 26
8.5. Assessment of Safety 28
8.5.1. Laboratory examinations 28
8.6. Adverse event definitions and reporting 29
8.6.1. Adverse Event definition 29
8.6.2. Serious Adverse Event 29
8.6.3. Eliciting Adverse Event information 29
8.6.4. Adverse Event reporting period 30
8.6.5. Adverse Event reporting requirements 30
8.6.6. Grading of Adverse Event severity 30
8.6.7. Adverse Event causality assessment 31

8.6.8. Exposure in utero ... 31
8.6.9. Adverse event follow up ... 32

9. WITHDRAWAL CRITERIA ... 32

9.1. Rules in case of treatment interruption ... 32

9.2. Rules for permanently interrupting study treatment ... 32

10. DATA ANALYSIS AND STATISTICAL METHODS ... 33

10.1. Sample size determination ... 33

10.2. Definition of study populations included in the analysis ... 34

10.3. Subject Disposition ... 35

10.4. Baseline ... 35

10.5. Treatment Compliance ... 35

10.6. Efficacy Analysis ... 35

10.7. Safety Analysis ... 36

10.8. Analysis of other endpoints ... 36

10.9. Interim analysis ... 37

11. DATA SAFETY MONITORING BOARD ... 37

12. QUALITY ASSURANCE AND QUALITY CONTROL PROCEDURES ... 37

12.1. Investigator's file ... 37

12.2. Case report forms (CRFs) ... 38

12.3. Source documents ... 38

12.4. Record Retention ... 38

12.5. Monitoring ... 38

12.6. Audits and inspections ... 39

12.7. Data Management ... 39

12.8. Confidentiality of trial documents and subjects records ... 39

13. PROTOCOL AMENDMENTS ... 40

14. TERMINATION OF THE STUDY ... 40

15. ETHICS ... 41

15.1. Informed consent process ... 41

15.2. Ethical aspects of subject inclusion and study procedures ... 42

15.3. Ethical aspects of study treatments ... 42

15.4. HIV status and VCT ... 42

15.5. Patient costs ... 42

16. INSURANCE AND LIABILITY ... 42

17. REPORTING AND PUBLICATION ... 42

18. REFERENCES ... 44

APPENDICIES: EXAMPLE PATIENT INFORMATION AND CONSENT FORM ... 47

ABBREVIATIONS – GLOSSARY OF TERMS

AE	Adverse event
ALT	Alanine aminotransferase (SGPT)
ASK	Academic Medical Center/ Slotervaart Hospital/ KIT (Amsterdam)
AP	Alkaline Phosphatase
AST	Aspartate aminotransferase
CBC	Complete blood count
CRF	Case report form
DNDi	Drugs for neglected diseases initiative
IEC	Independent ethics committee
IED	Institute of Endemic Diseases (University of Khartoum, Sudan)
FDA	Food and Drug Administration
GCP	Good clinical practice
ICH	International Conferences on Harmonization
IED	Institute of Endemic Diseases
IM	Intramuscular
IV	Intravenous
KEMRI	Kenya Medical Research Institute (Kenya)
LEAP	Leishmania East Africa Platform
PCR	Polymerase chain reaction
PI	Principal investigator (see note Section 13)
SAE	Serious adverse event
SSG	Sodium stibogluconate
ULN	Upper limit of normal
VL	Visceral leishmaniasis
WBC	White blood cell
WHO	World Health Organization
WNL	Within normal limits

PROTOCOL SYNOPSIS

**Protocol Title**	A phase II randomized, parallel arm, open-labelled clinical trial to assess the safety and efficacy of the combination of SSG plus single dose AmBisome®, Miltefosine plus single dose AmBisome® and Miltefosine alone for the treatment of primary visceral leishmaniasis in Eastern Africa
**Phase**	Phase 2
**Indication**	Treatment of primary VL in East Africa
**Protocol Number**	LEAP 0208
**Background Information and Trial Rationale**	SSG for 30 days remains the mainstay of VL treatment in East Africa. AmBisome® at a total dose of 20-30 mg/kg has also been used in the field. There is only one study on the use of miltefosine in the region. All three drugs have been studied in, are registered and available in India.<br><br>Phase II and III combination trials involving AmBisome® and miltefosine have been done or are underway in India. However while efficacy to SSG is poor in India, it remains high in East Africa. Conversely, the minimum effective dose of AmBisome® is only 5mg/kg in India; while it is currently being investigated in an ongoing study and is anticipated from one completed trial and field use to be higher in East Africa. Paromomycin efficacy is also considerably lower in Africa compared to India.<br><br>The current study intends to look at potential feasible short course combination therapies as well as evaluate (and possibly register) miltefosine in its conventional dose against VL in Sudan and Kenya.

**Trial Objectives**	**Primary:**<br>• To assess the efficacy of the following treatments for primary VL at day 28:<br>- the combination of single dose AmBisome® and a 10 day course of SSG<br>- the combination of single dose AmBisome® and a 10 day course of Miltefosine<br>- Miltefosine<br><br>**Secondary**<br>• To assess the efficacy of the following treatments for primary VL at day 210:<br>- the combination of single dose AmBisome® and a 10 day course of SSG<br>- the combination of single dose AmBisome® and a 10 day course of Miltefosine<br>- Miltefosine<br><br>• To assess the safety up to day 60 of the following treatments for primary VL:<br>- the combination of single dose AmBisome® and a 10 day course of SSG<br>- the combination of single dose AmBisome® and a 10 day course of Miltefosine<br>- Miltefosine<br><br>• To assess the pharmacodynamics of the following treatments for primary VL:<br>- the combination of single dose AmBisome® and a 10 day course of SSG<br>- the combination of single dose AmBisome® and a 10 day course of Miltefosine<br>- Miltefosine<br><br>• To study the pharmacokinetics of Miltefosine alone and in combination with AmBisome® in both adult patients and paediatric patients.
**Trial Endpoints**	**Primary endpoints:**<br>• Initial cure: proportion cured at Day 28<br>**Secondary endpoint:**<br>• Final cure: proportion cured at day 210 (6 months follow up)<br>• Adverse events and serious adverse events occurring in the three study arms up to day 60<br>• Description of the pharmacodynamic properties of all 3 arms<br>• Description of the pharmacokinetic properties of miltefosine

**Trial Design**	A phase II randomized, parallel arm, open-labeled clinical trial to assess the safety and efficacy of the combination of SSG plus single dose AmBisome®, Miltefosine plus single dose AmBisome® and Miltefosine alone for the treatment of primary visceral leishmaniasis in Eastern Africa.
**Main Entry Criteria**	**Inclusion criteria:**<br>1) Patients with clinical signs and symptoms of VL and diagnosis confirmed by visualization of parasites in tissue samples (lymph node, bone marrow or spleen where relevant) on microscopy.<br>2) Patients aged between 7 (to allow for blood sampling) and 60 years (inclusive) who are able to comply with the protocol.<br>3) Patients for whom written informed consent has been signed by the patients themselves (if aged 18 years and over) or by parents(s) or legal guardian for patients under 18 years of age.<br>4) HIV negative status<br>Patients who fulfill the above inclusion criteria will be enrolled in the study.<br><br>**Exclusion criteria:**<br>1) Patients who have received any anti-leishmanial drugs in the last 6 months / relapse cases.<br>2) Patients with a negative lymph node/bone marrow (or spleen) smears.<br>3) Patients with severe protein and or caloric malnutrition (Kwashiorkor or marasmus; Adults: BMI ≤15, Children W/H<70, presence of oedema)<br>4) Patients with previous history of hypersensitivity reaction to SSG or Amphotericin B.<br>5) Patients suffering from a concomitant severe infection such as TB or any other serious underlying disease (cardiac, renal, hepatic) which would preclude evaluation of the patient's response to study medication.<br>6) Patients suffering from other conditions associated with splenomegaly such as schistosomiasis.<br>7) Patients with previous history of cardiac arrhythmia or an abnormal ECG<br>8) Patients who are female of child bearing age (all females who have achieved menarche)/ pregnant or lactating.<br>9) Patients with haemoglobin < 5gm/dl.<br>10) Patients with WBC < 1 x $10^3/mm^3$.<br>11) Patients with platelets < $40,000/mm^3$.<br>12) Patients with abnormal liver function (ALT and AST) tests of more than three times the normal range.<br>13) Patients with serum creatinine outside the normal range for age and gender.<br>14) Major surgical intervention within 2 weeks prior to enrollment.
**Study Duration**	Approximately 18 months
**Test Drugs**	SSG, AmBisome®, Miltefosine
**Statistics**<br>Sample size<br>Randomisation<br>Summary of analysis	The study was designed and will be analyzed according to group-sequential methods, specifically the triangular test. Maximum sample size per arm will be 63 subjects. Randomisation will be in pre-determined blocks that will be undisclosed to the investigators. Sequential analysis will be done every 45 patients (15 per arm) using the Triangular Test with the following parameters (p_0 =0.75, p_a=0.9, α=0.05, β =0.05) as explained in section 10.

1. Background and Study Rationale

Visceral leishmaniasis (VL) is the most serious of all the leishmaniases as it is fatal if not treated. Even with the available treatments, up to 15% mortality is common in remote areas. VL is caused mainly by *Leishmania donovani* in Sub-Saharan Africa. Not all infected people develop clinical (symptomatic) VL. Some people, particularly those living in transmission areas may be partially immune and have a sub-clinical infection only, which spontaneously resolves, but until resolution occurs they may be a reservoir of infection in the community. The incubation period ranges from 3 weeks to over 2 years, with an average of 2-4 months. The disease is characterized by fever, weight loss, hepatosplenomegaly, lymphadenopathy, anaemia, leucopenia and thrombocytopenia. An estimated 500,000 new cases of VL occur annually and ninety percent of cases occur in five countries: Bangladesh, Brazil, India, Nepal and Sudan. Recent epidemics claimed thousands of lives in Sudan. Treatment failure is becoming a common problem in endemic areas.

In children, the high prevalence of malnutrition, anaemia and subsequent impaired immunity increase the likelihood that infection will progress to clinically evident, symptomatic disease. Concomitant acute infections such as malaria, tuberculosis and pneumonia compound the problem. Infected adults may also suffer these problems and the additional burden of HIV co-infection, particularly in Ethiopia.

A complication of visceral leishmaniasis, particularly in Sudan, is post kala-azar dermal leishmaniasis (PKDL) which usually occurs in the months following treatment in people who have recovered from VL. Such a complication will be monitored for during the course of the study.

Gedaref State is one of the main VL foci in Sudan. Others are Sennar, Blue Nile, Upper Nile and Unity provinces. The endemic localities in Gedaref State are located within the region bounded by Rahad River in the south and west; and Atbara River in the North-East; the region also bordering Ethiopia in the east. The catchment areas of White and Blue Nile rivers in southern Sudan (areas from Naser and Malakal in the south up to Dinder - south of Rahad River) are also important VL foci. Isolated foci (secondary foci) are known in North Darfur, South Kordofan, and to a limited extent in the province of Equatoria. One of the study sites (Dooka) will recruit patients from the Gedaref foci.

Main VL endemic areas in Kenya include East Pokot (Kachileba), Baringo and Wajir. The second study site will recruit patients principally from the Baringo area. The disease is also endemic in Ethiopia (mainly northwest Ethiopia in the lowlands of Metema and Humera, southwest Ethiopia in the Segen, Woitu and Omo river basins, and in other isolated foci in the Rift Valley), Uganda (West Pokot region), Eritrea (the Red Sea littoral localities of Nakfa, Afabet, Algena, Keren and the district of Teseney, North of Humera) and Somalia (Bakool region).

The available monotherapeutic treatment options in East Africa are far from satisfactory as they are either expensive (AmBisome®) or toxic (Sodium stibogluconate, SSG) with emerging parasite resistance. Conventional monotherapies also require prolonged treatment durations (1 month) and have

further issues around safety and compliance.

To overcome these limitations, alternative combination treatment protocols need to be explored. Combination therapies using drugs that have different modes of action, like SSG, liposomal amphotericin B (henceforth called AmBisome®) and Miltefosine could provide an ideal combination for treatment with reasonable cost (due to reduction of dosages needed). SSG is a pentavalent antimony-carbohydrate complex that was developed more than 80 years ago. Its mechanism of action remains elusive. m-chlorocresol, which is included in the sodium stibogluconate formulation as a preservative contributes to its anti-leishmanial activity. It is postulated that the apparent decrease in ATP and GTP synthesis contributes to decreased macromolecular synthesis and to decreased *Leishmania* viability. It is also suggested that inhibition of glycolysis and the citric acid cycle may partially explain the inability to phosphorylate ADP. AmBisome® affects sterol biosynthesis, disrupting the parasite membrane. The liposomal formulation allows for effective penetration and sustained tissue concentrations, low plasma levels and hence diminished toxicity with high efficacy. Miltefosine (hexadecylphosphocholine) a phosphorylcholine ester of hexadecanol, which is a membrane-active alkylphospholipid, is the first effective oral agent for VL. Miltefosine acts as a potential inhibitor of enzymes involved in membrane lipid metabolism (e.g. de novo synthesis of phosphatidylcholine) and through numerous potential interactions with cell membrane components and cell signalling pathways. It also induces apoptosis possibly through mitochondrial dysfunction or a causal relationship with phosphatidylcholine biosynthesis.

SSG is either included in the National drug lists or is registered in Sudan, Ethiopia and Kenya. Registration is also pending in Uganda. AmBisome® is used as rescue medication for VL in Sudan and Ethiopia. There is limited experience of use of Miltefosine in East Africa except in one trial in Ethiopia which demonstrated a comparable efficacy and lower case fatality rate to SSG.[1] It is not registered in the region. All 3 drugs have been registered in India and phase IV for miltefosine has been completed.[2,3] A phase II study in India has recently been published demonstrating potential efficacy of the combination of AmBisome® and Miltefosine.[4] A phase III trial sponsored by DNDi exploring this and other combinations is currently underway in India. SSG monotherapy remains efficacious in Africa and it has been given in the field as an effective short (15 day) course in Sudan.[5,6] In India, AmBisome® 5mg/kg given once has over 90% efficacy.[7] From field use and one clinical trial, a higher minimum dose is already anticipated in Africa and a trial to evaluate the minimum effective monotherapy dose of AmBisome® is shortly to commence in Ethiopia and Sudan.[8,9] The small phase II clinical trial in Kenya noted that 10 out of 10, 9 out of 10 and only 1 out of 5 was cured by total doses of 14mg/kg, 10mg/kg and 6mg/kg respectively.[9] This study here intends to look at potential feasible short course combination therapies as well as evaluate Miltefosine in its conventional dose against VL in Sudan and Kenya. Children will be included due to the fact that the study population in Sudan and Kenya are largely paediatric (60-70%) and that the test drugs have already been tested and used in a paediatric VL population in monotherapy and in adults in combination (AmBisome® & Miltefosine).[4,10] High doses of AmBisome® (multiples of 10mg/kg) have also been used to treat immunocompromised patients with fungal infections and can thus be considered safe when administered as a single dose (see investigators brochure).

2. Study Objectives and Endpoints

The objective of this study is to promote the geographical extension of currently available VL treatments and develop new, short course combination treatments in East Africa. Miltefosine has been developed and is registered for VL in India and several other countries. However it is not registered anywhere in East Africa. Little is also known of its pharmacokinetic properties in VL patients or in a paediatric population, and there are no such data in the East Africa region. Short course combination treatment is currently being developed by DNDi in India and shows promise of reducing treatment to around 10 days. DNDi intends to purse a similar strategy in East Africa with the intention of first doing a proof of concept phase II study for efficacy and safety of two potential combinations.

2.1. Objectives

2.1.1. Primary Objective

To assess the efficacy of the following treatments for primary VL at day 28:

- the combination of single dose AmBisome® and a 10 day course of SSG
- the combination of single dose AmBisome® and a 10 day course of Miltefosine
- Miltefosine

2.1.2. Secondary Objectives

- To assess the efficacy of the following treatments for primary VL at day 210:
    - the combination of single dose AmBisome® and a 10 day course of SSG
    - the combination of single dose AmBisome® and a 10 day course of Miltefosine
    - Miltefosine

- To assess the safety up to day 60 of the following treatments for primary VL:
    - the combination of single dose AmBisome® and a 10 day course of SSG
    - the combination of single dose AmBisome® and a 10 day course of Miltefosine
    - Miltefosine

- To assess the pharmacodynamics of the following treatments for primary VL:
    - the combination of single dose AmBisome® and a 10 day course of SSG
    - the combination of single dose AmBisome® and a 10 day course of Miltefosine

- Miltefosine

- To study the pharmacokinetics of Miltefosine alone and in combination with AmBisome® in both adult patients and paediatric patients

Following this trial, it is anticipated that data collected on miltefosine will be used to submit registration dossiers in the region. Pharmacokinetic data will allow assessment on the elimination half life and other pharmacokinetic properties of the drug in the local population in both monotherapy and combination and hence assess whether the drug can be practically used in women of child bearing age.

2.2. Study Endpoints

For efficacy, the primary endpoint (initial cure at day 28) will be based on a clinical assessment and parasitological evaluation (e.g. bone marrow or lymph node for Sudan and bone marrow or spleen aspirate for Kenya). The secondary endpoint (final cure at day 210) will be a clinical evaluation. Parasitology will be done only if clinically indicated according to a standardised clinical assessment. As the sample size will be calculated according to initial (day 28) cure, this assessment has been chosen as the primary endpoint.

2.2.1. Primary Endpoint

- Initial cure: proportion cured at Day 28

2.2.2. Secondary Endpoint(s)

- Final cure: proportion cured at day 210 (6 months follow up)
- Adverse events and serious adverse events occurring in the three study arms up to day 60
- Description of the pharmacodynamic properties of all 3 arms
- Description of the pharmacokinetic properties of miltefosine

3. Study design and study design rationale

3.1. Study design

A phase II randomized, parallel arm, open-labeled clinical trial to assess the safety and efficacy of the combination of SSG plus single dose AmBisome®, Miltefosine plus single dose AmBisome® and Miltefosine alone for the treatment of visceral leishmaniasis in Eastern Africa.

Partners involved in the study are as follows:
Institute of Endemic Diseases, University of Khartoum, Sudan: protocol development, implementation and recruitment site (Dooka)
Kenya Medical Research Institute: data management, recruitment site (Baringo)
ASK [Academic Medical Center/ Slotervaart Hospital/ KIT (Koninklijk Instituut voor de Tropen)], Amsterdam: pharmacokinetics/ pharmacodynamics

London School of Hygiene & Tropical Medicine: statistical support
Leishmania East Africa Platform: technical support and coordination
Drugs for Neglected Diseases Initiative: sponsor

3.2. Study duration and duration of subject participation

There will be two recruitment sites for this study- the Professor EL-Hassan treatment centre in Dooka, Gedaref, Sudan and KEMRI, Kenya. Approximately two thirds of patients will be recruited from Dooka, Sudan, and one third from Kenya. The overall study duration is expected to be 18 months, from recruitment to final assessment. At least 9 months is estimated for the recruitment period.

Treatment arms in the combination arms will last for 11 days, while miltefosine monotherapy will be administered for the conventional 28 days. All patients will be hospitalized for around 30 days (including day 0). The primary endpoint will occur at day 28 (day 1 being the first day of treatment) or the day after treatment ceases in the event in a delay of the treatment period (e.g. interruption of miltefosine monotherapy treatment). If well, a patient will be discharged home after the primary endpoint. An additional assessment will occur at 60 days (one month after primary endpoint). The final secondary endpoint will occur at the day 210 (6 months after the primary endpoint).

3.3. Rationale of study design

The development of miltefosine has already been completed in India and the drug is registered in several countries. Very little is known about the pharmacokinetics of the drug in VL patients, with no data from Africa. A recent study by Dorlo et al done on Dutch soldiers suggested a 2 compartment model with a first elimination half-life of 7.05 days and a terminal half-life of 30.9 days.[11] If verified in this different patient population, a possible implication could be that the current recommendation of contraception for 3 months may be insufficient, meaning less feasible use of the drug in women of child bearing age. Till this can be determined, this group has therefore been excluded from the study. Therefore the objective of including a miltefosine monotherapy arm is to collect pharmacokinetic as well as further regional safety and efficacy data to facilitate registration. The dose of miltefosine chosen follows the recommended dose established in clinical trials in India.

Both combination arms are essentially exploratory arms to determine whether either arm shows sufficient promise regarding safety and efficacy to merit further evaluation in a phase III comparative study whence recommendations can be made. The choice of a single dose AmBisome® with 10 days of a partner drug would represent a significant reduction in the current treatment length of 28/30 days and in potentially reducing toxicity. It could also result in considerable savings from long hospitalisation, reduction in (opportunity) costs to patients and further encourage health seeking behaviour.

The rationale to combine miltefosine and AmBisome® is based on completed phase II (where a single shot of AmBisome® 5mg/kg and miltefosine 2.5mg/kg for 7 days has an efficacy of 98%) and ongoing phase III studies in India. [4] These combined drugs seem to show activity enhancement or synergy. An *in-vivo* study by Seifert et al demonstrated an activity enhancement index (AEI) of 11.3 with amphotericin B

deoxycholate plus miltefosine compared to miltefosine alone, indicating a mechanistic interaction between the two drugs.[12] Whether there is also a pharmacokinetic interaction between the two drugs, maybe underlying the mechanistic interaction, remains unknown and therefore, amongst other reasons, it was decided to also implement pharmacokinetics of miltefosine in this study.

The rationale of combining SSG and Ambisome is due to the good efficacy of the former drug. In a study in Africa (LEAP 01014A), a complete case analysis of 115 patients on conventional dose SSG (20mg/kg for 30 days) showed an efficacy of 93% (DNDI unpublished data). This is in contrast to India where efficacy has declined to as low as 35%. Paromomycin has not been considered due to its relatively poor efficacy in the region, particularly in Sudan (LEAP 0104 A, DNDI unpublished data).

The dosage of Ambisome® was determined according to data from both Asia and Africa. Studies in India have determined that a single dose of 5mg/kg has a 91% efficacy.[4] However a small phase II study in Kenya suggested that 6mg/kg (with 1 out of 5 cured) is not sufficient in the East-African context. Total doses recommended in the region for Ambsiome® is around 20mg/kg, and field data confirm this as an appropriate dosage in monotherapy.[8] For this reason an appropriate dosage for combination was estimated to be more than 5mg/kg, less than the total dose required (20mg/kg), and within a range where good safety data exists on a safe single dose (5-10mg/kg). Therefore a single dose of 10mg/kg was chosen for this study.

SSG dose and duration has been chosen based on its efficacy in the region, though toxicity remains an issue. Small field evaluations confirm that 20mg/kg given for only 15 days is efficacious.[5,6] Therefore in combination, duration of 10 days has been estimated to be adequate.

With miltefosine monotherapy, the dose and duration used is based on the above data from India, and the one phase III trial conducted in Africa showing a 6 month cure rate of 93% in the non-HIV VL patients.[1] For combination therapy, a similar 10 day regimen was therefore anticipated. The advantage of an AmBisome® and miltefosine combination could be a single dose of the former given in hospital, with the latter completed as out patient oral treatment

The design of the study is based on a similar exploratory phase II combination trial done in India by Sundar et al.[4] A triangular design has been chosen in order to identify at the earliest point within the designated power an arm that is either efficacious (benchmarked at 90%) or non-efficacious (threshold of 75%). This will include repeated analyses after every 45 (15 per arm) patients recruited (see section 10). If at any sequential analysis, an arm is either shown to be efficacious or non efficacious within the set statistical parameters, it will be stopped. The maximum number of sequential analyses will be five and the maximum sample size will be 189. The primary endpoint has been chosen to allow such an evaluation at an early point in time. If the conventional 6 months (day 210) follow up after assessment for the primary endpoint (day 28) had been chosen, a large sample size may already be recruited. However, as the latter is still an important endpoint to base a final decision on whether to take a test arm into phase III, it has been included as a secondary

endpoint. Parasitological assessment at end of treatment will remain as the benchmark; however for day 210, the method employed in Indian VL clinical trials of using a clinical assessment (with parasitology done only if clinically indicated) will be followed. This is to avoid painful invasive procedures such as bone marrow aspirate on subjects who are well.

Safety assessments will include haematological, renal and hepatic monitoring and will be done at regular time points (day 0, 7, 14, 28) to ensure safety is adequately assessed, including an early assessment at day 3 to evaluate for nephrotoxicity. An additional follow up at 60 days (32 days after assessment of the primary endpoint) will be included to allow a further safety assessment one month after treatment ends/ the previous assessment, as well as additional necessary pharmacokinetic evaluation. It is also expected that early treatment failures can be caught within this time frame.

Figure 1- Overall study design

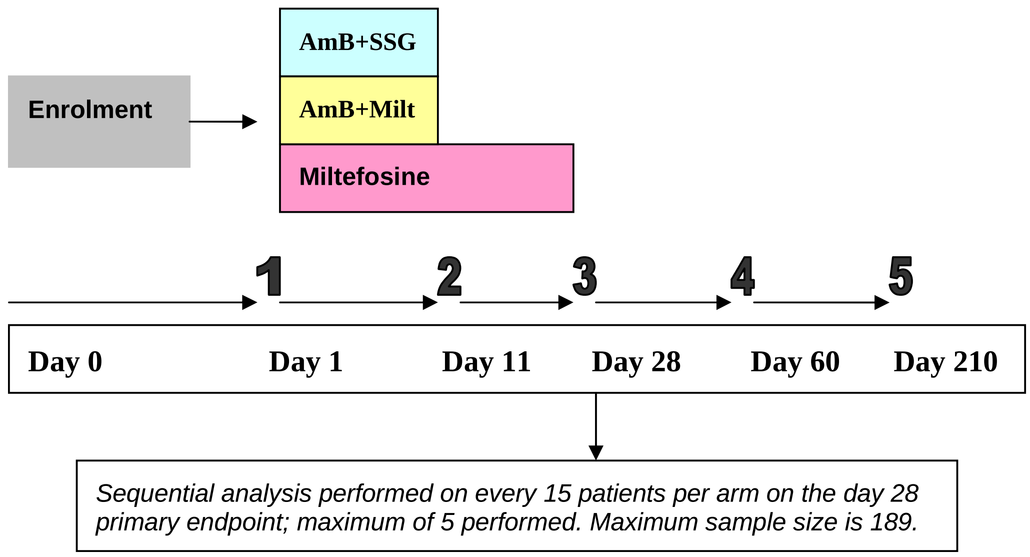

O= Assessment and informed consent
1= Commencement of treatment
2= End of combination treatment regimens (day 11)
3= Primary efficacy endpoint at day 28 (miltefosine arm ends day 28)
4=Further safety and follow up assessment at day 60
5= Final assessment at 6 months after day 28 primary endpoint (day 210)

4. Selection of Subjects

A maximum of 189 subjects will be enrolled into this study. The following eligibility criteria were designed to select subjects for whom the protocol treatment is considered appropriate. All relevant medical and non-medical conditions will be taken into consideration when deciding whether this protocol is suitable for a particular subject. Eligibility criteria will not be waived by the investigator. Any questions

regarding a subject's eligibility will be discussed with DNDi Medical Coordinator prior to subject's enrollment.

4.1. Inclusion criteria

Subjects must meet **all** of the following inclusion criteria to be eligible for enrollment into the study:

- Patients with clinical signs and symptoms of VL and diagnosis confirmed by visualization of parasites in tissue samples (lymph node, bone marrow or spleen where relevant) on microscopy.
- Patients aged between 7 (to allow for blood sampling) and 60 years (inclusive) who are able to comply with the protocol.
- Patients for whom written informed consent has been signed by the patients themselves (if aged 18 years and over) or by parents(s) or legal guardian for patients under 18 years of age.
- HIV negative status

Patients who fulfill the above inclusion criteria will be enrolled in the study.

4.2. Exclusion criteria

The presence of any of the following will exclude a subject from study enrolment:

- Patients who have received any anti-leishmanial drugs in the last 6 months/ relapse cases.
- Patients with a negative lymph node/bone marrow (or spleen) smears.
- Patients with severe protein and or caloric malnutrition (Kwashiorkor or marasmus ; Adults: BMI $\leq$15, Children W/H<70, presence of oedema)
- Patients with previous history of hypersensitivity reaction to SSG or Amphotericin B.
- Patients suffering from a concomitant severe infection such as TB or any other serious underlying disease (cardiac, renal, hepatic) which would preclude evaluation of the patient's response to study medication.
- Patients suffering from other conditions associated with splenomegaly such as schistosomiasis.
- Patients with previous history of cardiac arrhythmia or an abnormal ECG
- Patients who are female of child bearing age (all females who have achieved menarche) / pregnant or lactating.
- Patients with haemoglobin < 5gm/dl.
- Patients with WBC < 1 x 10^3/mm^3.
- Patients with platelets < 40,000/mm^3.
- Patients with abnormal liver function (ALT and AST) tests of more than three times the normal range.
- Patients with serum creatinine outside the normal range for age and gender.
- Major surgical intervention within 2 weeks prior to enrolment.

<u>NB</u>

Relevant tests will be done to exclude the above listed conditions.

Patients with common VL co-Infections, particularly respiratory tract infections and

malaria will be treated for these conditions first before commencement of study medication. The latter can be commenced 48 hours after completion of co-infection treatment. Cases of severe malaria and serious lower respiratory tract infections should be excluded (concomitant severe infections).

5. Schedule of events (*Table 1*)

**Protocol Activities and Forms to Be Completed**	**Screening day 0: may last more than one day**				**Treatment (day)**																			**At Follow-up after day 30†**	
	0	-1	-2	-3	1	2	3	4	5	6	7	8	9	10	11	12	13	14	→	21	→	28		**Day 60**	**Day 210**
Consent form & Screening form & HIV test	X																								
Clinical assessment	X										X							X		X		X		X	X
Lymph node/Bone marrow/ Spleen aspiration	X																					X		X ††	X ††
rK39 dipstick	X																								
ECG	X										X							X							
Urinalysis	X						X											X							
Complete Blood count*	X										X							X				X		X	X
Urea, Creatinine & serum electrolytes (Na^+, K^+ and Mg^{2+})**	X						X				X							X				X		X	X
Liver function Test**	X										X							X				X			
Quantitative PCR (measure response)*	X						X				X							X				X		X	X
Assessment for PKDL (or para kala azar DL)	X																					X		X	X
SSG (IV/IM) [20 mg/Kg/day]						X	X	X	X	X	X	X	X	X	X										
AmBisome (IV) in combination					X																				
Miltefosine (oral) in combination arm [2.5 mg/Kg/day]						X	X	X	X	X	X	X	X	X	X										
Miltefosine alone arm [2.5 mg/Kg/day]					X	X	X	X	X	X	X	X	X	X	X	X	X	X	→	X	→	X			
PK study for Miltefosine – Arm 2 – *Adults****						X#		X			X				X									X	X
PK study for Miltefosine – Arm 2 – *Children****						X#					X				X									X	X
PK study for Miltefosine – Arm 3 – *Adults****					X#		X				X							X		X		X		X	X
PK study for Miltefosine – Arm 3 – *Children****					X#						X							X				X		X	X
Volume of blood (ml) taken from Group 2- children	7.5					2.5	2.5				10.5				0.5			10				7.5		7.5	7.5
Volume of blood (ml) taken from Group 3- children	7.5				2.5		2.5				10.5							10.5				8		7.5	7.5

Clinical Clinical assessments include pulse, blood pressure, temperature, weight, height (at baseline), spleen size, liver size.

† Patients will be asked to return for an assessment between day 60 and 210 in the event in the event of any medical problems. Blood count, biochemistry, liver function tests, parasitology and PK analysis will be done in the event of such a visit. At the day 210 visit, flexibility (of +/- 21 days) will be allowed for timing due to practical difficulties patients may face to meet exact timing of visits.

†† Parasitology will only be done in the follow up period after day 28 if clinically indicated (i.e. if reappearance of symptoms and signs of VL).

*2.5 mls of EDTA blood for Complete blood count; and only 0.2 mls EDTA blood used for PCR ** 7.5 ml blood for urea, creatinine, liver function test.

*** The PK study will be done on the specified days with 2.5 mls of EDTA blood taken before/ after and 0.5 mls during treatment (the use of dried blood spots is currently under investigation).

On first day of Miltefosine treatment, blood samples will be drawn 3 times: just prior to first dose, 4 & 8 hours post first-dose to assess the absorption-phase of miltefosine (total volume needed 2.5 mls). All other PK samples during treatment will be taken prior to the morning dose.

Volumes of blood required for children are shown in bottom two rows. In total, 41.5 mls are required in the first 1 month during admission in group 3 and 41 mls for group 2.

6. Enrolment procedures

All subjects who fulfill the study criteria will be consented and entered into the study. Patients will be randomized in predefined blocks by the DNDi data centre (based in KEMRI, Nairobi). The site investigators will be blinded to the size of these blocks. Randomization will be stratified by center (Sudan and Kenya). Randomization codes will be prepared by the data centre in sealed sequentially numbered, opaque envelopes and will be under the control of the site investigator. The study will be un-blinded due to the considerable differences in the treatment arms.

7. Treatments

7.1. Investigational Product

Name: **Liposomal Amphotericin B (AmBisome®)**

Class: a macrocyclic, polyene antifungal antibiotic produced by *Streptomyces nodosus*.

Mechanism of action: affects sterol biosynthesis, disrupting the parasite membrane.

Commercial source: Gilead.

Product appearance: AmBisome® comes as a sterile lyophilised powder in a 15ml sterile Type 1 clear glass vial containing a yellow powder with the active ingredient amphotericin B 50mg encapsulated in liposomes. The closure consists of a butyl rubber stopper and aluminium ring seal with a removable plastic cap. Vials are packed in cartons of 10, with 10 filters provided.

Administration: each vial is reconstituted with 12ml sterile water (yielding 4mg/ml amphotericin B) and given as an intravenous infusion in 5% dextrose. The infusion can be concentrated from 2.00mg to 0.20mg amphotericin B per ml.

Name : **Miltefosine (Impavido®)**

Class: Phosphocholine analogue.

Mechanism of action: interferes with the synthesis and metabolism of phospholipids. It may also interfere with the parasite's membrane signal transduction, and glycosylphosphatidylinositol anchor biosynthesis.

Commercial source: Paladin labs.

Product appearance: comes as 10mg and 50mg capsules as a pack of 28 or 56 capsules sealed in 4 or 8 aluminium blister stripes, each containing 7 capsules.

Administration: oral, at a dose of 2.5mg/ kg daily for 28 days.

Name: **Sodium Stibogluconate**

Class: Pentavalent antimonial.

Mechanism of action: decrease in ATP and GTP synthesis contributes to decreased macromolecular synthesis and to decreased *Leishmania* viability.

Commercial source: Albert David; distributed by International Dispensary Association.

Product appearance: A faintly straw coloured fluid in a multi-dose brown opaque vial of 30ml containing SSG BP equivalent to 100mg pentavalent antimony in each ml

(total 3g).
Administration: Given IV (slow over 5 minutes) or IM at a dose of 20mg/kg body weight for up to 30 days.

7.2. Doses and treatment regimens

Dosing and duration of the study drugs is as follows:

- AmBisome® will be given as single dose at day 1 at a dose of 10 mg/kg body weight infused in 5% dextrose running for 1-2 hours.
- Miltefosine will be given orally at a dose of 2.5 mg/kg body weight daily, up to maximum total dose of 150mg, for 28 days when used alone.
- Miltefosine will be given orally at a dose of 2.5 mg/kg body weight daily up to maximum total dose of 150mg, for 10 days starting at day 2 following a single dose of AmBisome® 10mg/Kg.
- SSG will be given IV/IM at a once daily dose of 20 mg/kg body weight daily without ceiling for 10 days starting from day 2 following a single dose of AmBisome®.

Therefore dose regimens will be as follows:
Arm 1: AmBisome® one dose of 10mg/kg body weight (IV) on day 1 followed by 10 days of SSG at 20mg/kg body weight (IV/IM) from days 2-11
Arm 2: AmBisome® one dose of 10mg/kg body weight (IV) on day 1 followed by 10 days Miltefosine at 2.5mg/kg body weight (oral) from days 2-11
Arm 3: Monotherapy course of Miltefosine at 2.5mg/kg body weight (oral) from days 1-28

AmBisome® (IV) and SSG (IM/IV) will be prepared and administered by trained nursing staff. Miltefosine treatment will be given as directly observed treatment daily to ensure compliance. All treatments will be given under the supervision of the trial physician. Miltefosine capsules will be administered with meals. Doses of Miltefosine may be given once or twice daily depending on dosage; e.g. subjects requiring two or more 50mg capsules of miltefosine per day will have their total daily dose divided into 2 individual doses to be taken morning and evening. A table with daily dosing according to weight is included below.

***Table 2:** showing dosing of miltefosine capsules according to weight ranges.*

Weight range (kg)	Total approximate dose (2.5mg/kg)	Morning	Evening
10.0-13.9	30	10mg x 2	10mg x 1
14.0-17.9	40	10mg x 2	10mg x 2
18.0-21.9	50	50mg x 1	-
22.0-25.9	60	50mg x 1	10mg x 1
26.0-29.9	70	50mg x 1	10mg x 2
30.0-49.9	100	50mg x 1	50mg x 1
≥50.0	150	50mg x 2	50mg x 1

7.3. Drugs labelling, packaging, accountability

Commercially available products will be used as all drugs have been registered for VL (e.g. India). Trial specific labelling will be applied prior to use. All study medications will be kept in a locked room that can be accessed only by the pharmacist, designated trial charge nurse or the investigator. The study medications will not be used for other purposes other than this protocol. Under no circumstances will the investigator or site staff supply study medications to other investigators or sites, or allow the medications to be used other than as directed by this protocol without prior authorization from DNDi. Adequate records on storage conditions (e.g. daily temperature logs), receipt, use, return, loss, or other disposition of medication will be maintained.

7.4. Storage

AmBisome®: The product should be stored at 25°C or below, not frozen or exposed to light. The shelf life is 3 years. Once reconstituted, chemical and physical in-use stability has been demonstrated for 24 hours at 25+/-2°C in vials exposed to ambient light. This is increased to 7 days if stored at 2-8°C. However to avoid contamination, once reconstituted, the product should be stored at 2-8°C and be used within 24 hours.

Miltefosine: The product should be stored in the original package and protected from moisture. The shelf life is 5 years. Packaging should be undamaged prior to use.

SSG: The product should be stored below 30°C and protected from light. The shelf life is 3 years. The contents should not be used for more than one month after opening.

7.5. Blinding and procedures for un-blinding

This is an un-blinded study.

7.6. Concomitant medications

Concomitant medications should be avoided unless absolutely necessary. Conditions such as malaria and respiratory tract infections should be treated prior to VL treatment if identified early enough. Exclusion of such common conditions should be undertaken for all VL patients prior to commencement of treatment. However it is expected that some patients may require management of such common conditions during study treatment. Drugs that prolong the QT interval (e.g. quinine) should be avoided while SSG is being administered. If it is urgently required to give such drugs, SSG should be stopped; otherwise alternatives should be sought (e.g. artemisinins). AmBisome is incompatible with saline and should not be mixed with other drug or electrolyte solution during administration. For miltefosine, there are no known interactions with other commonly used medications, though this cannot be excluded.

8. Study Assessments

8.1. Timing of Assessments

Assessments will be timed at day 0, 3, 7, 14, 21, 28, 60 and 210 and will include clinical, parasitology, haematology, biochemistry and pharmacokinetic assessments. Additional pharmacokinetic assessments will be done on day 1, 2,4,11 and 21. Full

details of the schedule are in section 5. Maximum total volumes of blood to be taken over the whole period of the study will be around 55 mls for children and 60 mls for adults. As day 60 and 210 assessments will require some flexibility on dates due to patient travel, visit windows for each will be as follows- day 60 (+/- 10 days) and day 210 (+/- 21 days).

8.2. Baseline Assessments

Baseline assessments will include anthropometric, clinical and laboratory evaluations. Symptoms and signs of the disease will also be recorded in the source documents and CRF.

8.3. Assessment of Efficacy

Primary efficacy endpoint

The primary efficacy endpoint will be a clinical and parasitology assessment (lymph node/ bone marrow/ spleen aspirate). Subjects which have made a clinical improvement and have full parasitological clearance at day 28– test of cure (TOC) - will be considered as an (initial) treatment success. For the sequential analysis, all patients who have parasites visible at day 28 will be considered treatment failures.

Secondary efficacy endpoint

The secondary efficacy endpoint will be a clinical (and if indicated a parasitology) assessment at day 210 (6 months post treatment). Treatment success will include subjects with no relapse (requiring rescue treatment) up to day 210, as assessed by clinical status- in other words patients must have no symptoms or signs of the disease. If symptoms and signs of disease are present (e.g. fever, enlargement of spleen, weight loss, anaemia), a parasitology assessment will be done to confirm treatment failure.

Slow Responders

A slow responder is a subject with a parasitaemia of 2+ and above at pre-treatment who achieves a good response during treatment and who is clinically well, but who does not completely clear parasites by day 28 (a drop in parasitaemia, but scanty parasites are visualised; e.g. +1). These subjects do not require rescue medication. They will be monitored at the day 60 evaluation where a repeat parasitology will be performed. In the event that this parasitology assessment is positive, the subject will be given rescue medication and be considered a treatment failure. In the event that the subject clears parasites and is clinically well, no rescue medication is given and the day 210 evaluation will be performed. If patients remain clinically well at this stage, they will be considered as a treatment success at the secondary endpoint.

Rescue medication

The decision to give rescue medication will be based on a standard guideline for all trial sites. Any patient who receives rescue medication at any point will be considered a treatment failure. Patients will be given rescue medication for 4 reasons:

i) failure to respond to initial anti-leishmanial treatment during the first 28 days

ii) failure to tolerate trial medication / occurrence of adverse event(s) during receipt of test drugs that requires (treatment) withdrawal

iii) recurrence of symptoms, signs and presence of parasites after day 28 (treatment failure)

iv) or development of severe para or post kala azar dermal leishmaniasis (PKDL) that necessitates rescue treatment.

Rescue treatment given includes:

- AmBisome 30mg/kg IV split into multiple doses (according to country protocol: Sudan- 3mg/kg/day for 10 days)
- SSG 20mg/kg IM for 30-60+ days: for patients not responding to initial rescue treatment or for patients requiring treatment for severe PKDL.

PKDL

Patients will be monitored closely for para-kala-azar dermal leishmaniasis and PKDL through the course of the study. Diagnosis will be made clinically based on the typical appearance and distribution of the rash. Presence of Para or PKDL will be noted at the day 0, 28, 60 and 210 assessments. Grading will also be noted during the assessment times as follows: mild (Grade 1), moderate (Grade 2) and severe (Grade 3). A patient with grade-1 Para or PKDL has lesions mainly on the face and head, with others scattered on arms, chest and back. When the lesions affect parts of the body, including the hands and feet, the case is considered grade 3. All other cases, with lesions on the head, scalp, forearms, upper legs and upper chest but not the hands and feet, are considered grade 2. The most severe form is grade 3 with mucosal and/or eye involvement.[13] A patient with this form will be defined as requiring rescue medication.

8.3.1. Assessments performed

Clinical assessment

The clinical evaluation will involve measuring the spleen size by palpation below the left costal margin, liver size, temperature, pulse, blood pressure, body weight on days 0, 7, 14, 21, 28, 60 and 210. Height will be measured at baseline only. Daily progress during treatment will be documented by the trial medical staff. This will include monitoring for adverse events which will be documented according to section 8.6.

8.3.2. Laboratory examinations

Parasitology will be done on day 0 (baseline), day 28 and day 60 and 210 if clinically indicated. It will involve a lymph node or bone marrow aspirate in Sudan or a spleen or bone marrow aspirate in Kenya. Aspirates are smeared on slides, stained and graded according to the standard logarithmic criteria.

***Table 3**: Parasitology grading scale: number of parasites visualised by high powered field.*

Count Oil Immersion x 100	
**6+**	> 100,000/1000
**5+**	10,001-100,000/1000
**4+**	1,001-10,000/1000
**3+**	101-1,000/1000
**2+**	11-100/1000
**1+**	1-10/1000
**0**	0/1000

8.4. Other Assessments: Pharmacokinetic evaluation of miltefosine and pharmacodynamic evaluation of all treatments

Pharmacokinetics

The effect of a systemic disease like VL on the pharmacokinetics of miltefosine remains largely unknown. The only extensive pharmacokinetic data available are from a relatively healthy patient group suffering from cutaneous leishmaniasis (CL) published by one the collaborating groups, ASK. Data here indeed indicated a two compartment disposition model with a long terminal elimination half-life which has significant implications on the use of this drug in a monotherapy (with an increased risk of emergence of resistant strains) and in women of child bearing age.[11]

A phase I/II trial in a paediatric population with VL in India indicated a ~9% lower per protocol efficacy of miltefosine (2.5 mg/kg) in children than in adults.[14,15] However, a larger paediatric phase II trial could not replicate these findings and found an equivalent efficacy between paediatric and adult patients.[16] Whether the observed difference in efficacy and differences between paediatric trials are due to a difference in pharmacokinetics or higher variability in pharmacokinetics in children, is unknown; there has never been a thorough pharmacokinetic study of miltefosine in VL patients, let alone in paediatric VL patients. Therefore, the population pharmacokinetic parameters (and their intra- and inter-individual variability) of miltefosine in both children and in adults will be assessed. In children a more sparse sampling strategy will be applied and also alternative, less invasive, sampling methods are currently under investigation to be applied in this paediatric group.

Miltefosine will be given after a single dose of AmBisome® in treatment Arm 2 (see Test Drugs). From a theoretical point of view, pharmacokinetic interactions can be expected between the liposomal sphere itself and the phospholipid-like miltefosine (e.g. scavenging of miltefosine by the liposomal structure, thereby increasing miltefosine's central elimination half-life). To assess whether there is any pharmacokinetic interaction between the two test drugs, the pharmacokinetics of miltefosine will be investigated in this study both in the monotherapy and combination arms. The schedule of pharmacokinetic assessments are highlighted in section 5.

Miltefosine plasma concentrations will be analyzed in both treatment groups where miltefosine is included (Arm 2 and Arm 3). Bioanalysis of miltefosine will be done on a maximum of 250 µL of plasma samples or dried-blood spots (currently under

investigation) using a validated liquid chromatography tandem mass spectrometry (LC-MS/MS) method for miltefosine.[17]

Technical details:

Plasma concentrations of miltefosine will be determined as recently described by an LC-MS/MS assay.[17] In brief, the assay consists of a solid-phase extraction on BondElut PH cartridges (Varian Inc., Bergen op Zoom, The Netherlands) containing 100 mg sorbent, using maximally 250 ml of a plasma sample (samples taken during treatment will only need 50 µl of plasma sample). Cartridges are conditioned with 1 ml of acetonitrile and 1 ml of 0.9 M acetic acid in water (pH 4.5). After sample loading and washing with 50% (vol/vol) methanol in water, miltefosine will be eluted with 0.1% (vol/vol) triethylamine in methanol. The eluate containing the analyte will be kept in autosampler vials at a temperature of 10°C and injected directly on a Gemini C18 column (150 mm by 2.0-mm inner diameter; 5-mm particle size) (Phenomenex, Torrance, CA) in combination with a guard column (Gemini C18 precolumn; 4.0 mm by 2.0-mm inner diameter) (Phenomenex), both of which will be operated at ambient temperature. The analyte will be eluted from the analytical column by using an isocratic elution with a mixture of 10 mM aqueous ammonium hydroxide and 10 mM ammonium hydroxide in methanol (at a ratio of 5:95), with a total run time of 7 min.

Detection will be performed by MS/MS with electrospray ionization, using an API 2000 mass spectrometry system (Sciex, Thornhill, Ontario, Canada) with Analyst software (version 1.2). Miltefosine will be monitored in the positive-ion mode, with the following transition of precursor ([MH]+) to product ion: m/z 408.4 to 124.8. The quantifiable range of the assay is 4 to 2,000 ng/ml miltefosine in plasma. The assay is validated over this range according to FDA guidelines for the validation of bioanalytical assays. At the lowest level (4 ng/ml), the intra-assay precision is lower than 10.7%, the inter-assay precision is 10.6%, and accuracies are between 95.1 and 109%. At higher concentrations, the assay performs even better in terms of precision and accuracy.[17] Samples with a concentration above the upper limit of quantification are diluted in drug-free human control EDTA-plasma to fit the calibration curve. Along with study samples, a calibration curve will be prepared and analyzed in duplicate, together with a set of quality control samples at low, mid, and high levels, prepared and analyzed in triplicate.

The applicability of alternative sampling strategies which are less invasive, especially for children, is currently being investigated and evaluated in a human volunteer study by the bioanalytical laboratory that is involved in this study (ASK). Using non-linear pharmacokinetic modeling (NONMEM) a population pharmacokinetic model will be made in the two different treatment groups and differences in these parameters between adults and children will be evaluated.

To assess the effect of VL on the pharmacokinetics of miltefosine, the population pharmacokinetic model derived from this study will be compared with the previously published population pharmacokinetic model from the CL patient group.

Pharmacodynamics

This study will also use a real time quantitative reverse transcriptase polymerase chain reaction (qRT-PCR) for *L. donovani* in EDTA blood based on the amplification of single-stranded 18S rRNA sequences without the interference of DNA, to assess treatment response during and after treatment and as a possible test of cure in patients with VL.[18] Differences in parasite clearance in the blood are to be expected between the treatment arms: combinations are likely to result in a more rapid elimination of *Leishmania*, hence their decreased duration of treatment. By following parasite clearance with qRT-PCR during treatment, we hope to decipher whether there are underlying pharmacodynamic differences between the two combination therapies and the miltefosine monotherapy, which, in contrast, cannot be detected by comparing final cure rates.

In treatment arms 2 & 3 (arms receiving miltefosine), the outcome and parasite clearance measured by qRT-PCR will be linked to miltefosine pharmacokinetics. Modeling of miltefosine PK-PD in our patient will enable to establish variability in pharmacokinetics in relation to outcome (pharmacodynamics) which can be seen as an essential component in the development of new treatment regimens.

Measurement of *Leishmania* parasite loads in the blood by PCR has been successfully used in the control of VL-HIV coinfections (http://www.ajtmh.org/cgi/reprint/75/5/858.pdf).[19] In this trial the blood parasite counts will be used as a pharmacodynamic indicator of parasite clearance rate and thus as a measure of response to treatment. Intra- and inter-individual variations will accurately be estimated and disentangled with non-linear mixed effects modelling.

For this purpose, a volume of approximately 0.2 mls EDTA whole blood will be immediately separated from blood samples taken (e.g. for Complete Blood Count) on day 0, 3, 7, 14, 28, 60 and 120 at the study site. The sample will be immediately diluted with DNA stabilizing buffer solution (L6 buffer) and can then be stored up to 2 years at frozen temperature. The qRT-PCR will be performed by the Department of Parasitology at the Royal Tropical Institute, Amsterdam.

Samples taken for both PK and PD will be sent for analysis in Amsterdam only for the purposes stated in this protocol.

8.5. Assessment of Safety

Clinical Assessment

The clinical evaluation will involve measuring the spleen size, liver size, temperature, pulse, blood pressure, body weight on days 0, 7, 14, 21, 28, 60 and 210. ECG will be performed on days 0, 7, 14. Daily progress during treatment will be documented by the trial medical staff. This will include monitoring for adverse events which will be documented according to section 8.6.

8.5.1. Laboratory examinations

Haematological and biochemical assessment

Blood will be analysed for haemoglobin, WBC, platelets, urea, creatinine, serum electrolytes (Na^+, K^+, Mg^{2+}), and liver function (AST, ALT, ALP, bilirubin) tests on days 0, 3 (biochemistry only), 7,14, 28, 60 and 210.

Urinalysis

Dipstick analysis will be performed on days 0, 3, 14, and 28.

8.6. Adverse event definitions and reporting

8.6.1. Adverse Event definition

An adverse event will be defined as any untoward medical occurrence (any unfavourable and unintended sign, symptom or disease, including an abnormal laboratory finding) in temporal association with the use of the investigational treatment and may or may not be causally related to it.

Abnormal laboratory (hematology and biochemistry) results will be reported as adverse events if the abnormality occurs or worsens after institution of the study treatment, and if they require clinical intervention or further investigation, unless they are associated with an already reported clinical event.

8.6.2. Serious Adverse Event

An adverse event will be defined as serious if it is

- fatal
- life-threatening
- requires or prolongs hospitalization
- results in persistent or significant disability
- is a congenital anomaly/birth defect
- results in an important medical event that may not be immediately life threatening or does not directly result in death or hospitalization, but which may jeopardize the patient or may require intervention to prevent the other outcomes listed above

Serious events also include any other event that is defined as serious for the specific purposes of the protocol or which is defined as serious by the regulatory agency in the country in which the event occurred

8.6.3. Eliciting Adverse Event information

The investigator is required to report all directly observed adverse events and all adverse events spontaneously reported by the trial subject using concise medical terminology. In addition, each trial subject will be questioned about the occurrence of adverse events on a daily basis during the hospitalisation period (day 1-28), and in follow up periods (day 60 and 210) with a generic question such as “have you/has your child felt different in any way/ had any problems since starting the new treatment/the last assessment?” If the response is “Yes”, the nature of the event, the date and time (where appropriate) of onset, the duration, maximum intensity (see

below) and relationship to treatment will be established (see below). Details of any dosage/schedule modification or any corrective treatment will be recorded on the appropriate pages of the CRF.

8.6.4. Adverse Event reporting period

The adverse events reporting period for this trial begins

- Upon administration of the first dose of trial medication for non-serious events
- Upon administration of the first dose of study medication for serious events

and ends at day 60 when a further safety evaluation occurs. This is 32 days after the last dose of study medication for the longest treatment arm (miltefosine). Note that miltefosine has a half life of around 7 days.

All adverse events that occur during the adverse event reporting period specified in the protocol must be reported to DNDi, whether or not the event is considered medication related. In addition, any adverse event that occurs subsequent to the adverse event reporting period that the investigator assesses as possibly related to the investigational medication should also be reported as an adverse event.

8.6.5. Adverse Event reporting requirements

Information on adverse events must be evaluated by a physician. Each adverse event is to be classified by the investigator as serious or non-serious. This classification will determine the reporting procedure for the event.

All serious adverse events (SAE) are to be reported immediately (within 24 hours of awareness of SAE by the investigator) to the DNDi medical coordinator, using the SAE report form. This includes a description of the event, onset date and type, duration, severity, relationship to study drug, outcome, measures taken and all other relevant clinical and laboratory data. The initial report is to be followed by submission of additional information (follow-up SAE form) as it becomes available. Any follow-up reports should be submitted as soon as possible, and if possible within 7 working days.

Serious adverse events should also be reported on the clinical trial adverse event case report form (CRF). It should be noted that the form for reporting of SAE (SAE form) is not the same as the adverse event section of the CRF. Where the same data are collected, the two forms must be completed in a consistent manner, and the same medical terminology should be used.

Non-serious adverse events are to be reported on the CRF, which is to be submitted to DNDi as specified in the Study Documentation section of this protocol. In the CRF, a given adverse event will be recorded only one time per patient, and the severity recorded will be the maximum level reached. If several distinct episodes of the same condition occur, their number will be recorded in the CRF.

8.6.6. Grading of Adverse Event severity

The investigator will use the terminology MILD, MODERATE, or SEVERE to describe the maximum severity of the adverse event. This information will be entered in the adverse event case report forms. For purposes of consistency, these severity grades are defined as follows:

MILD	Does not interfere with subject's usual functions
MODERATE	Interferes to some extent with subject's usual functions
SEVERE	Interferes significantly with subject's usual functions

It is to be noted the distinction between severity and seriousness of adverse events. A severe adverse event is not necessarily a serious event.

In this study a specific adverse event severity grading scale will be used based on the National Cancer Institute Common Toxicity Criteria in order to standardize reporting between investigators and sites.

8.6.7. Adverse Event causality assessment

For both serious and non-serious adverse events, the investigator is required to assess the possible relationship between the adverse event and the study drug, i.e. to determine whether there exists a reasonable possibility that the study drug caused or contributed to the adverse event. Causality will be listed as not related, unlikely, possible or probable.

To help investigators with the decision binary tree in the evaluation of causality, the CIOMS VI group recommends that investigators be asked to consider the following before reaching a decision:

- Medical history
- Lack of efficacy/worsening of existing condition
- Study medications
- Other medications (concomitant or previous)
- Withdrawal of study medication, especially following trial discontinuation / end of study medication
- Erroneous treatment with study medication (or concomitant)
- Protocol related procedure

The decision to suspend, and resume treatment or to permanently interrupt treatment due to an adverse event will be left to the clinician in charge.

8.6.8. Exposure in utero

All women of child bearing age are to be excluded from this study. This will be done by screening all young women to see if menarche has commenced. This is specifically due to the teratogenic potential of miltefosine (demonstrated in rats). The manufacturer recommends that effective contraception should be used during and up to 3 months after treatment of miltefosine.

However, in the unlikely event that any trial subject becomes or is found to be pregnant while receiving an investigational drug or within 90 days of discontinuing the investigational drugs, the investigator must submit the event on an SAE form. This must be done irrespective of whether an adverse event has occurred. The information submitted should include the anticipated date of delivery.

The investigator will follow the subject until completion of the pregnancy or until pregnancy termination (i.e., induced / spontaneous abortion). The investigator will provide pregnancy outcome information as a follow up to the initial SAE form.

In the case of a live birth, a paediatrician should assess the infant at the time of birth and submit a report.

8.6.9. Adverse event follow up

All adverse events should be followed until they are resolved or the investigator assesses them as chronic or stable or the subject participation in the trial ends (i.e., until a final report is completed for that subject).

In addition, all serious adverse events and those non-serious events assessed by the investigator as possibly related to the investigational drug must continue to be followed even after the subject participation in the trial is over. Such events should be followed until they resolve or until the investigator assesses them as “chronic” or “stable.” Resolution of such events is to be documented on the CRF.

9. Withdrawal criteria

If a subject withdraws from the study, the reason must be noted on the CRF. If a subject is withdrawn from the study because of a treatment limiting adverse event, thorough efforts should be made to clearly document the outcome.

A subject should be withdrawn from the trial treatment if, in the opinion of the investigator, it is medically necessary, or if it is the wish of the subject. If a subject does not return for a scheduled visit, every effort should be made to contact the subject. In any circumstance, every effort should be made to document subject outcome, if possible.

If the subject withdraws consent, no further evaluations should be performed and no attempts should be made to collect additional data, with the exception of safety data, which should be collected if possible.

9.1. Rules in case of treatment interruption

In the event that treatment is interrupted (e.g. due to an adverse event), the decision to resume treatment will be taken by the site trial physician in conjunction with the PI and medical coordinator.

9.2. Rules for permanently interrupting study treatment

If a subject is withdrawn from the study before the full course of the treatment is completed, the physician must make all necessary arrangements to ensure that the subject receives the appropriate treatment for the relevant medical condition (e.g. with drug/s currently recommended by the national policy).

10. Data Analysis and Statistical Methods

10.1. Sample size determination

Miltefosine for 4 weeks has an efficacy at 6 months that exceeds 90%. In combination, Miltefosine for ten days is postulated (and suggested through a phase 2 trial in India) to be effective (over 90% efficacy) when combined with single dose AmBisome®. SSG for 15 days at a dose of 20 mg/kg/day has been shown to be effective in Sudan. In combination with AmBisome® it is postulated that 10 days of SSG treatment will be effective in more than 90% of patients.

The study was designed and will be analysed according to group-sequential methods specifically the triangular test. For ethical reasons, it has become common practice in clinical trials to perform interim analyses or use multistage designs to ensure that either harmful or ineffective treatments are discontinued as early as possible. However, even trials with planned interim analyses suffer from low power. Sequential analyses, on the other hand, are designed to allow for repeated testing throughout the trial recruitment period while maintaining good statistical properties (pre-specified type I and type II error) and reducing the necessary sample size. Sequential trials, where the data is analyzed after every patient is recruited, can be difficult to achieve in many settings. Group sequential trials were developed to allow for discrete data analysis after a pre-specified number of patients are recruited. The triangular test is one way of analyzing group sequential trials and uses straight line stopping boundaries. It involves analyzing the data as they accumulate, with points being plotted relative to a triangular region and stopping when the upper or lower boundary of the region is crossed (Graph 1). It has the advantage over other methods of analyzing group sequential trials, such as the discrete sequential probability ratio test, in that a closed continuation region is used ensuring a maximum sample size.[20,21]

Based on the rationale above, it will be assumed that a 28-day cure rate of 75% will be considered inadequate and such regimens would not be of interest for further investigation (p_0 =0.75). A 28-day cure rate of >90% would indicate adequate efficacy and recommend the regimen for further consideration (p_a=0.9). The type I error rate and power of the study were set to 5% and 95%, respectively (α=0.05, β =0.05). Based on these specifications, the boundaries of the test were calculated for the null hypothesis, H_0: $p \leq p_0$, and the alternative hypothesis, H_a: $p > p_a$ (see Graph 1). Data from each arm will be analyzed (for the evaluable patient population or per protocol analysis) after every 15 patients reach the primary endpoint of initial cure, where the actual values of Z and V will be calculated and then plotted on the graph. Depending on where the point falls, that arm of the trial will either continue collecting data for another 15 patients or will stop the trial concluding with a rejection of the null hypothesis (adequate efficacy) or a non rejection of the null hypothesis (inadequate efficacy). In the final analysis, asymptotic properties will be used to calculate unbiased estimators of the response rate and its 95% confidence interval taking into account the sequential nature of the analyses.[22]

When the analyses concludes that an arm should be stopped (i.e. a boundary has been crossed), the final cure rate (day 210) endpoint will also be evaluated. If the day 210 final cure is found to be <90%, then the arm (for arm 1 and 2) will not be considered for further study (e.g. phase 3).

Efficacy will also be calculated by centre though the study is not powered to detect potential differences between sites. However, it is estimated that Sudan will recruit twice as many patients as Kenya, and if a difference exists, a better efficacy is expected for Kenya. As Gedaref, Sudan is the main endemic zone of VL in Africa, this site will drive the overall response rate for the study.

Since we are using a sequential approach, the sample size is not defined or known in advance. The maximum sample size needed per arm would be 63 patients (189 total patients) and the maximum number of sequential analyses performed would be 5- at 15, 30, 45, 60 and 63 patients per arm (see Graph 1, upper horizontal axis). The actual sample size may be less than this, with its value depending on the actual proportion cured.

Graph 1: Triangular region for study arms showing the boundaries for analyzing the sequential trial using the Triangular Test, with the following parameters (p_0 =0.75, p_a=0.9, α=0.05, β =0.05, and n=15)

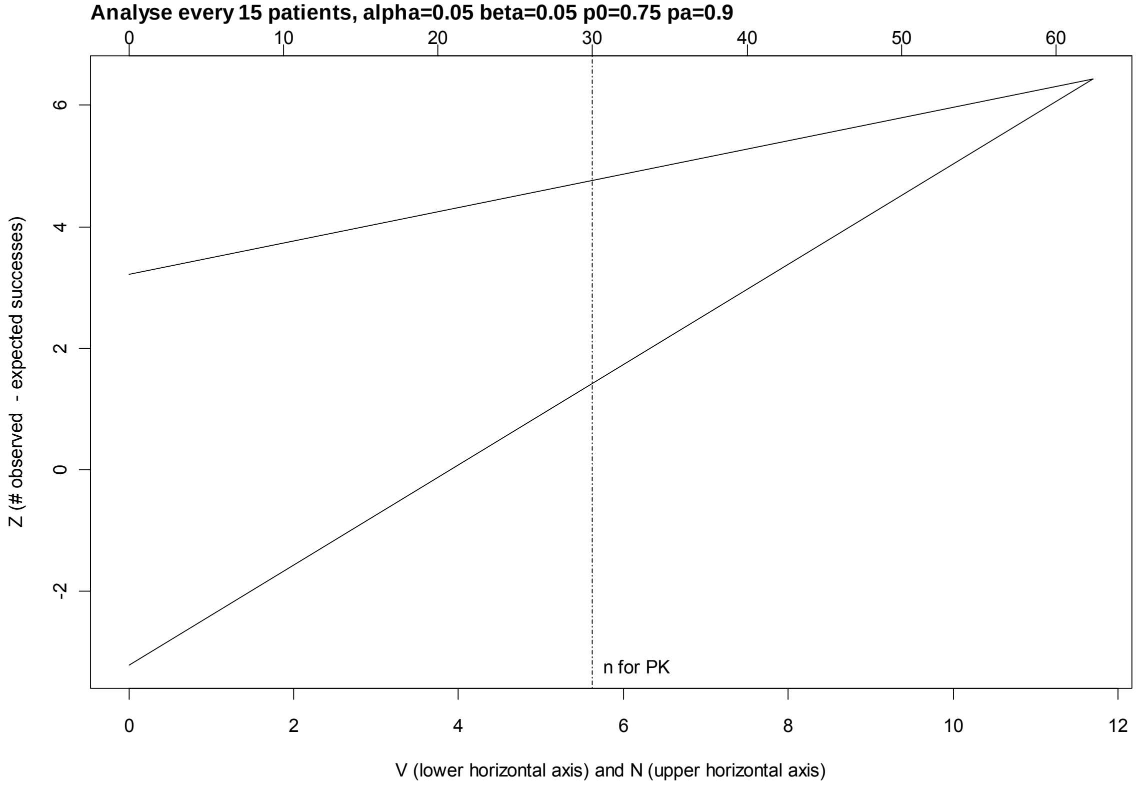

For the PK study, we aim for a sample size of at least 30 patients per arm for arm 2 and 3, unless efficacy is poor and the lower boundary is reached before this point. As three different groups are being assessed, it is possible that one group may be stopped prior to the others (e.g. in the event of low efficacy).

10.2. Definition of study populations included in the analysis

For the sequential and primary analysis (day 28), an intention to treat analysis will be

performed. For the secondary analysis an intention to treat, per protocol, and evaluable case analysis will be preformed.

10.3. Subject Disposition

All subjects entering the recruitment sites will be screened for eligibility and their consent sought as appropriate. Patient flow will be monitored and reported accordingly. No active recruitment for other clinical trials on primary VL will be ongoing during the recruitment phase of this study.

10.4. Baseline

Routine baseline characteristics including age, sex, height, weight, body mass index (for adults), weight for height nutritional score (for children), and symptoms (fever, weight loss, epistaxis, etc) will be documented as appropriate.

10.5. Treatment Compliance

All test drugs will be directly administered (IV/IM) or be directly observed (oral) by study medical staff. All failures with compliance (e.g. vomiting) will be appropriately document.

10.6. Efficacy Analysis

The primary and secondary endpoint have been described in section 8.3.

The primary endpoint (day 28) analysis will be defined as follows:

- <u>Treatment success</u>: absence of parasites on day 28 with clinical improvement; hence no rescue medication provided up to this time-point.
- <u>Treatment failure</u>: presence of parasites at day 28 *or* requirement of rescue medication due to lack of clinical improvement/ response *or* withdrawn from the study (e.g. due to a treatment limiting adverse event/ patient absconded) *or* death.

The data will be analysed as proportions according to an intention to treat analysis for each treatment arm.

The secondary endpoint (day 210) analysis will be defined as follows:

- <u>Treatment success</u>: absence of signs and symptoms of VL with no requirement for rescue medication at any time up to this final assessment. Note that patients who are parasitology positive on day 28 but are deemed as slow responders *and* who subsequently are clinically well and parasite free on the day 60 assessment may fall into this category (unless at any time between day 60 - day 210 they are deemed to require and are given rescue medication).
- <u>Treatment failure</u>: presence of signs of symptoms of VL, confirmed by presence of parasites in a parasitological investigation. It is mandatory that all patients who have possible presence of the disease have a parasitological assessment (e.g. bone marrow aspirate in Sudan, or bone marrow/ spleen aspirate in Kenya).

The data will be analysed as proportions per treatment arm according to either an

intention to treat, per protocol and evaluable analysis (to take into account missing data or missed assessment, in which case last known assessment will be used).

10.7. Safety Analysis

All patients randomized and who have been administered the first dose of study medication will be included in the safety analysis. Adverse events will include all events occurring between day 1 and day 60. Safety data will be summarized according the treatment group, each also by treatment period. Adverse event classification will be based on the NCI/ CTC criteria (January 2005 guidelines) and listed according to MedDRA coding. Non serious adverse events will be summarized, listed, presented as drug and non drug reactions for each arm. Serious adverse events will be summarized and compared using proportions per arm. All SAEs will be reported to and reviewed by the external DSMB according to current DNDI/ LEAP SOPs, who will advise on appropriate action. If more than 2 treatment related SAEs are reported in any one treatment arm that arm will be considered for cessation (after discussion with the DSMB).

10.8. Analysis of other endpoints

Pharmacokinetics

Miltefosine plasma concentrations will be determined in all samples collected for pharmacokinetics. If the predose concentration (just before the first dose of miltefosine) is less than or equal to the lower limit of quantitation, the subject's data without any adjustments can be included in all pharmacokinetic measurements and calculations. If the predose value is greater than the lower limit of quantitation, the subject will be dropped from the analysis.

Population pharmacokinetic-pharmacodynamic modeling will be performed using the nonlinear mixed-effects modeling program NONMEM, version VI (GloboMax LLC, Hanover, MD). Different pharmacokinetic models (various single and multi-compartment models) will be tested and adequacy of the models will be evaluated using statistical and graphical methods. The objective function value (equal to -2*log likelihood) provided by NONMEM will be used as a goodness-of-fit characteristic to discriminate between the different nested models, making use of the log likelihood ratio test.

Graphical model evaluation will be performed with the R-based model building aid Xpose (version 4, http://xpose.sourceforge.net/) and Perl speaks NONMEM (PsN): e.g. plots of conditional weighted residuals will be used for graphical inspection of the goodness of fit of the model.

The following primary individual Bayesian pharmacokinetic parameters will be estimated using the POSTHOCC option in NONMEM: absorption rate (k_a), volume(s) of distribution (*V/F*), clearance (elimination clearance [CL/*F*] or intercompartmental clearance [Q/*F*]); *V*, CL and Q are relative to bioavailability (*F*) because bioavailability is unknown. Secondary parameters like elimination half-life will be estimated from these primary parameters.
Both inter-individual variation and residual variation (which comprises the intra-individual variation) in the pharmacokinetic parameters will be estimated by the

appropriate error model. Standard errors for all pharmacokinetic parameters will be calculated with the COVARIANCE option in NONMEM.

Pharmacodynamics
Parasite loads will be quantified in the blood of patients during and after treatment, as a quantitative measure of response to therapy in all the treatment arms. Individual parasite clearance rates in the blood of patients in all three treatment arms will be modeled using NONMEM.

A semi-mechanistic indirect response model will be assumed here to model precisely the effect of the antileishmanial drugs on the kinetics of parasitemia. The pharmacodynamic response will be measured at a different site (blood) than the supposed site of action (liver, spleen and bone marrow) of the drugs in the body; therefore an appropriate transition model will be sought.

Discriminations between nested models will be made as described above for the pharmacokinetic model. For the treatment arms receiving miltefosine (arm 2 and 3), POST-HOC individual pharmacokinetic parameter estimates from the pharmacokinetic model for miltefosine will be fixed in the pharmacodynamic modeling, functioning as the driving force for the observed antiparasitic effect of miltefosine.

Parasite loads in blood, the kinetics of these parasite loads and the amount of time needed to get under the limit of detection, will be compared to the assessments of efficacy (primary and secondary endpoints) and the individual predictive value of the qRT-PCR during and after treatment will be evaluated to investigate the possible routine use of this non-invasive technique as a replacement for the currently used highly invasive spleen and bone marrow aspirates.

10.9. Interim analysis

Sequential analysis will be performed in this study as described in section 10.1.

11. Data Safety Monitoring Board

A Data Safety Monitoring Board (DSMB), composed of at least 3 members independent of the investigator and sponsors, will be set up prior to study initiation and will be composed of at least one member from Sudan and one from Kenya and one from a LEAP member country. The DSMB monitors the study in order to ensure that harm is minimised and benefits maximised for the study subjects. They will review the study data at pre-determined intervals and issue recommendations about the study. The form of data to be reviewed and intervals will be agreed prior to or soon after the study initiation and documented in the DSMB Charter.

12. Quality Assurance and Quality Control Procedures

12.1. Investigator’s file

The investigator must maintain adequate and accurate records to enable the conduct of the study to be fully documented and the study data to be subsequently verified. These documents include Investigator’s Site File, subject clinical source documents

and screening / enrolment logs. The Investigator's Site File will contain the Investigator brochures, protocol/protocol amendments, CRF and query forms, IEC and regulatory approval with correspondence, sample informed consent, drug accountability records, staff curriculum vitae and authorization forms and other appropriate documents/correspondence etc.

12.2. Case report forms (CRFs)

Data will be collected by laboratory technicians, medical doctors, clinical officers and nurses authorized by the investigator. It will be supervised by the Investigator and signed by the investigator or by an authorised staff member. Study-specific information will be entered into the Case Report Form (CRF). Data that are derived should be consistent with the source documents or the discrepancies should be explained. All CRF data should be anonymised; i.e. identified by study patient number and patient initials only.

The investigator at each trial site should ensure the accuracy, completeness, legibility, and timelines of all data reported to the sponsor in the CRFs and any other additional information that is required. The investigator is responsible for keeping all consent forms, screening forms, CRF and the completed subject identification code list in a secure location.

12.3. Source documents

The verification of the CRF data must be by direct inspection of source documents. Source documents include subject hospital/clinic records, physician's and nurse's notes, appointment book, original laboratory reports, ECG, X-ray, pathology and special assessment reports, signed informed consent forms, consultant letters, and subject screening and enrolment logs.

The investigator must maintain source documents such as laboratory and consultation reports, history and physical examination reports, etc., for possible review and/or audit by DNDi and/or Regulatory Authorities. The Investigator / designee will record the date of each subject's visit together with a summary of their status and progress in the study.

12.4. Record Retention

The investigator must keep all study documents on file for at least 15 years after completion or discontinuation of the study. After that period of time the documents may be destroyed with prior permission from DNDi, subject to local regulations.

Should the investigator wish to assign the study records to another party or move them to another location, DNDi must be notified in advance.

12.5. Monitoring

Monitoring visits to the trial site will be made periodically by DNDi representatives or designated clinical monitors to ensure that GCPs and all aspects of the protocol are followed. Source documents will be reviewed for verification of consistency with data on CRFs. The investigator will ensure direct access to source documents by DNDi or

designated representatives. It is important that the investigators and their relevant personnel are available during the monitoring visits.

The investigators will permit representatives of DNDi and/or designated clinical monitors to inspect all CRFs, medical records, laboratory work sheets and to assess the status of drug storage, dispensing and retrieval at anytime during the study. The corresponding source documents for each subject will be made available provided that subject confidentiality is maintained in accord with local regulations. The inspections are for the purpose of verifying the adherence to the protocol and to ensure the study is conducted according to GCP. It is important that the investigators and other trial site staff are available at these visits.

The monitoring visits provide DNDi with the opportunity to evaluate the progress of the study, verify the accuracy and completeness of CRFs, resolve any inconsistencies in the study records, as well as to ensure that all protocol requirements, applicable regulations, and investigator's obligations are being fulfilled. Four visit types are planned: pre-study, study start, during the study, and study end. Visits may also be performed by regulatory authorities.

It will be the clinical monitor's responsibility to inspect the CRF at regular intervals throughout the study, to verify the adherence to the protocol and the completeness, consistency and accuracy of the data being entered on them. The investigator agrees to cooperate with the clinical monitor to ensure that any problems detected in the course of these monitoring visits are resolved.

12.6. Audits and inspections

The trial site may also be subject to quality assurance audits by DNDi or designated representatives and/or to inspection by regulatory authorities or Independent Ethics Committees (IEC).

It is important that the investigators and their relevant personnel are available for possible audits or inspections.

12.7. Data Management

The CRF will be divided into several sections corresponding to the timing of assessments and will sent on an on-going basis to the data centre, based in KEMRI, Nairobi to allow sequential analyses as required. CRFs and source documents will be monitored by the clinical monitor. Discrepancies noted either by the monitor or data centre will be queried to the site PI. The trial data will be stored in a computer database maintaining confidentiality in accordance with national data legislation.

In order to ensure data quality, a uniform copy CRF will be designed for use for all sites. Data will then be sent to the data centre for data cleaning and analysis.

12.8. Confidentiality of trial documents and subjects records

The investigator must assure that subjects' anonymity will be maintained and that their identities are protected from unauthorized parties. On CRFs or other documents submitted to the sponsor, subjects should not be identified by their names, but

exclusively by an identification code. The investigator should keep a subject enrolment list showing codes, names, and addresses. The investigator should maintain documents for submission to sponsor authorized representative, and subject's signed written consent forms, in strict confidence.

13. Protocol Amendments

The Principal investigator will ensure that the study protocol is strictly adhered to throughout, and that all data are collected and recorded correctly on the CRF. The Principal investigator may contact the medical coordinator for a protocol waiver for minor deviations from the protocol, e.g. patient unable to attend during visit window.

All protocol modifications must be documented in writing. Any protocol amendment must be approved and signed by the sponsor and the Principal investigator and is to be submitted to the appropriate IEC for information and approval in accordance with local requirements, and to regulatory agencies if required. Approval by IEC (and Regulatory Authority, if applicable) must be awaited before any changes can be implemented, except for changes necessary to eliminate an immediate hazard to trial subjects, or when the change involves only logistical or administrative aspects of the trial, such as change in clinical monitor[s], change of telephone number[s].

The protocol amendment can be initiated by either sponsor or by any Principal investigator. The investigator will provide in writing the reasons for the proposed amendment and will discuss with the medical coordinator and sponsor.

14. Termination of the Study

Both the sponsor and the investigator reserve the right to terminate the study at any time prior to inclusion of the intended number of subjects, but they intend to exercise this right only for valid scientific or administrative reasons. Should this be necessary, both parties will arrange the procedures on an individual study basis after review and consultation. In terminating the study, the sponsor and the investigator will assure that adequate consideration is given to the protection of the subject's interest.

Reasons for termination by the sponsor(s) may include but not be limited to:

- Too low enrolment rate.
- Protocol violations.
- Inaccurate or incomplete data.
- Unsafe or unethical practices.
- Questionable safety of the test article.
- Suspected lack of efficacy of the test article.
- Following the recommendation of the DSMB or IEC
- Administrative decision.

Reasons for termination by the investigator may be:

- Insufficient time or resource to conduct the study
- Lack of eligible patients

In the event that a study is terminated either by the sponsor or by the investigator, the investigator has to:

- Complete all CRFs to the greatest extent possible
- Return all test articles, CRF, and related study materials to the sponsor who provided them
- Answer all questions of the sponsors or their representatives related to data of subjects enrolled at the site prior to study termination
- Ensure that subjects enrolled in the study who had not yet reached a follow up time point are followed up with the necessary medical care.
- Provide in writing the reasons for his decision to the national health authority and the sponsor.

15. Ethics

The experimental protocol for this study has been designed in accordance with the general ethical principles outlined in the Declaration of Helsinki and ICH guidelines for Good Clinical Practice (International Committee for Harmonization). DNDi assures that it will comply with all applicable state, local and foreign laws for protecting the rights and welfare of human subjects. This protocol and any protocol amendments will be reviewed / approved by an IEC before its implementation.

It is the responsibility of the Investigator to apply for review to the IEC of the country where the study takes place regarding local rules and regulations. Written approval from all involved IECs must be obtained before implementation of any protocol-specified intervention /investigation provided to the subject [such as subject information sheets or descriptions of the study].

Any modifications made to the protocol after receipt of the IEC approval must also be submitted by the investigator in writing to the IEC in accordance with local procedures and regulatory requirements.

15.1. Informed consent process

Inclusion in the study will occur only if the subject (for adults) or the parent/guardian (for children) gives written informed consent. It is the responsibility of the investigator / designee to obtain written informed consent from each individual participating in this study, after adequate presentation of aims, methods, anticipated benefits, and potential hazards of the study. The written informed consent document will be translated into the local language or a language understood by the subject(s). If needed, the person will be given time to discuss the information received with members of the community or family before deciding to consent. The subject or parent/guardian will be asked to provide written and signed consent.

If the subject is illiterate, a literate witness must sign (this person should have no connection to the research team, and, if possible, should be selected by the participant). The investigator should also obtain the assent of children (if appropriate), but their assent must be completed by the permission of a parent or guardian.

If new safety information results in significant changes in the risk/benefit assessment, the consent form should be reviewed and updated if necessary. All subjects (including those already being treated) should be informed of the new information, given a copy of the revised form and give their consent to continue in the study.

15.2. Ethical aspects of subject inclusion and study procedures

Inclusion and exclusion criteria follow previous LEAP studies. HIV co-infected patients are excluded due to the known impact this may have on efficacy. Study procedures are designed to ensure accurate assessment of efficacy while ensuring close follow up for safety- this includes the day 60 assessment that has been introduced to allow better evaluation of any adverse events that may occur after discharge from hospital and within 5 half lives of the study drugs. Blood or tissue samples taken from patients will not be used for any other purpose other than what has described in this protocol (or in future amendments) or a related protocol that receives a separate ethical approval from the local ethics committee.

15.3. Ethical aspects of study treatments

All drugs used have been studied and registered as monotherapies for VL. While treatments are of different lengths and administration, all patients will be hospitalised and followed up for similar periods of time.

15.4. HIV status and VCT

All patients will be offered counselling and screening for HIV (voluntary counselling and testing programme (VCT). This will be done at the same time as consent is obtained for inclusion in the trial. Patients who decline VCT or are found to be HIV positive will not be eligible to participate in this trial but will receive appropriate treatment, according to national treatment guidelines. Additionally, they will be referred onwards for treatment, surveillance and follow up according to the national protocol for HIV positive patients.

15.5. Patient costs

Patients will be reimbursed for travel to and from the study site but will not receive any payment for trial participation. Any medication that is required during the trial period will be provided free of charge to the patient. Food during the in-patient treatment phase will also be provided free of charge to the patient. This is seen as an essential part of the patient care plan bearing in mind the high prevalence of malnutrition and the poverty of these patients.

16. Insurance and Liability

DNDi is insured to indemnify the investigator against any claim for damages brought by a research subject who suffers from a research related injury during the performance of the trial according to the protocol.

17. Reporting and publication

The clinical trials will be registered with a recognised clinical trial registry specifically www.clinicaltrials.gov.

Before publication, all study results are considered confidential and shall not be made available to any third party by any member of the investigating team without an appropriate confidentiality agreement and/or written authorisation of the sponsor. It is anticipated that the results of this trial will be of sufficient medical importance to warrant publication(s) in an international peer-reviewed journal, and/or presentations at scientific meetings. In accord with standard editorial and ethical practice, the sponsor will generally support publication of multicentre trials only in their entirety and not as individual centre data. DNDi as sponsor will render all necessary assistance to the investigators to ensure this occurs in a timely manner. The investigator(s) agrees to submit all manuscripts or abstracts to the sponsor prior to submission, and allow sufficient time for review and input by sponsor and co-authors to ensure completeness and scientific accurateness. Authorship will be determined by mutual agreement.

References

1. Ritmeijer, K., et al. A comparison of miltefosine and sodium stibogluconate for treatment of visceral leishmaniasis in an Ethiopian population with high prevalence of HIV infection. *Clin Infect Dis*, 2006, 43(3): 357-64.

2. Bhattacharya, S.K., et al. Phase 4 trial of miltefosine for the treatment of Indian visceral leishmaniasis. *J Infect Dis*, 2007, 196(4): 591-8.

3. Sundar, S., et al. Oral miltefosine for Indian visceral leishmaniasis. *N Engl J Med*, 2002, 347(22): 1739-46.

4. Sundar, S., et al. New Treatment Approach in Indian Visceral Leishmaniasis: Single-Dose Liposomal Amphotericin B Followed by Short-Course Oral Miltefosine. *Clin Infect Dis*, 2008, 47(8): 1000-6.

5. Zijlstra, E.E, et al. Kala-azar in displaced people from Southern Sudan: epidemiological, clinical and therapeutic findings. *Trans R Soc Trop Med Hyg,* 1991, 85(3): 365-9.

6. Zijlstra, E.E, et al. The treatment of kala-azar in the Sudan with sodium stibogluconate: a randomized trial of three dosage regimens.*Trans R Soc Trop Med Hyg,* 1993, 87(3): 307-9.

7. Bern, C., et al. Liposomal amphotericin B for the treatment of visceral leishmaniasis. *Clin Infect Dis*, 2006, 43(7): 917-24.

8. Mueller, M., et al. Unresponsiveness to AmBisome in some Sudanese patients with kala-azar. *Trans R Soc Trop Med Hyg*, 2006, 100(4): 327-34.

9. Berman, J.D., et al. Efficacy and safety of liposomal amphotericin B (AmBisome) for visceral leishmaniasis in endemic developing countries. *Bull World Health Organ*, 1998, 76(1):25-32.

10. Singh, U.K., et al. Miltefosine in children with visceral leishmaniasis: a prospective, multicentric, cross-sectional study. *Indian J Pediatr*, 2006, 73(12): 1077-80.

11. Dorlo, T.P., et al. Pharmacokinetics of miltefosine in Old World cutaneous leishmaniasis patients. *Antimicrob Agents Chemother*, 2008, 52(8): 2855-60.

12. Seifert, K., Croft, S.L. In vitro and in vivo interactions between miltefosine and other antileishmanial drugs. Antimicrob Agents Chemother, 2006. 50(1): 73-9.

13. Musa, A. M., Khalil, E. A. G., Raheem, M. A., Zijlstra, E. E., Ibrahim, M. E., El-Hassan, I. M., Mukhtar, M. M. & El-Hassan, A. M. The natural history of

Sudanese post-kala-azar Dermal Leishmaniasis: Clinical, immunological and prognostic features. *Annals of Tropical Medicine and Parasitology*, 2002, 96: 65-72.

14. Sundar, S., et al. Oral miltefosine treatment in children with mild to moderate Indian visceral leishmaniasis. *Pediatr Infect Dis J*, 2003, 22(5): 434-8.

15. Sundar, S., et al. Miltefosine in the treatment of leishmaniasis: Clinical evidence for informed clinical risk management. *Ther Clin Risk Manag*, 2007, 3(5): 733–740.

16. Bhattacharya, S.K., et al. Efficacy and tolerability of miltefosine for childhood visceral leishmaniasis in India. *Clin Infect Dis*, 2004, 38(2): 217-21.

17. Dorlo, T.P., et al. Development and validation of a quantitative assay for the measurement of miltefosine in human plasma by liquid chromatography-tandem mass spectrometry. *J Chromatogr B Analyt Technol Biomed Life Sci*, 2008, 865(1-2): 55-62.

18. De Vries, P.J., et al. Quantification of the response to miltefosine treatment for visceral leishmaniasis by QT-NASBA. *Trans R Soc Trop Med Hyg*, 2006, 100(12): 1183-6.

19. Mary, C., et al. Reference values for Leishmania infantum parasitemia in different clinical presentations: quantitative polymerase chain reaction for therapeutic monitoring and patient follow-up. *Am. J. Trop. Med. Hyg.,* 2006, 75(5): 858–863,

20. Bellissant, E. Application of the triangular test to phase II cancer clinical trials. *Stats in Med*, 1990, Vol 9, 907-917.

21. Ranque, S., et al. Triangular test applied to the clinical trial of azithromycin against relapses in *Plasmodium vivax* infections. *Malaria Journal*, 2002, 1:13.

22. Bellissant, E., et al. The group sequential triangular test for phase II cancer clinical trials. *American Journal of Clinical Oncology*, 1996, 19(4): 422-30.

Appendices

1. PATIENT INFORMATION AND CONSENT
2. ADVERSE EVENT GRADING TO BE USED FOR STUDY
3. CHANGES INTRODUCED TO PROTOCOL
4. BUDGET

PATIENT INFORMATION AND CONSENT FORM

TITLE:

"A phase II randomized, parallel arm, open-labeled clinical trial to assess the safety and efficacy of the combination of sodium stibogluconate plus single dose AmBisome®, Miltefosine plus single dose AmBisome® and Miltefosine alone for the treatment of primary visceral leishmaniasis in Eastern Africa. "

EXAMPLE PATIENT INFORMATION AND CONSENT FORM

Form 1:	***For patients of age 18 and above***

SPONSOR: Drugs for Neglected Diseases initiative (DNDi), Geneva, Switzerland

Contact persons

1. Dr. Ahmed Mudawi
   Dooka Hospital

2. Dr Monique Wasunna
   KEMRI

3. [Chairs, National Ethics Review Committee, and Institutional Ethics Review Committee, (Sudan and Kenya)]

Important notice!

This patient information and consent form is to be read in the language that the patient understands. Therefore, please ask the patient for their preferred language. This form is available in English and Arabic

PART 1. INFORMATION SHEET

Principal Investigators

Prof E Khalil Institute of Endemic Diseases, University of Khartoum, Sudan

Dr Ahmed Musa Institute of Endemic Diseases, University of Khartoum, Sudan

Dr Monique Wasunna Kenya Medical Research Institute

Sponsor: Drugs for Neglected Diseases *initiative* (DNDi), Geneva, Switzerland

Introduction

We are studying visceral leishmaniasis (also called kala azar) - a fatal disease if untreated, which is common in our country. The tests you have had performed confirm that you are suffering from this disease.

We are studying different ways of giving the 3 drugs called AmBisome®, sodium stibogluconate and miltefosine to treat this disease, and we would like you to participate in this study. These drugs alone have been shown to be very effective in treating visceral leishmaniasis either in many countries. In this study, we plan to compare different ways of giving these drugs. Firstly we want to establish that one of the drugs, miltefosine works in the same way it has been demonstrated to in other countries like India. Secondly, we intend to give the drugs in combination with each other- for instance by giving AmBisome® and sodium stibogluconate and AmBisome® and miltefosine together for 10 days. At the end of this trial, we hope that a treatment regimen, which is safe, effective and shorter than the usual treatment duration will be available. This trial is expected to last 18 months, and we intend to enrol up to 189 patients. With your permission, we would like to include you in this trial. Your participation will be for 7 months. If you do not give consent to participate in this study you will be treated with sodium stibogluconate – which is the standard drug for treatment of the disease in this country.

Procedures during the trial

As explained above, there are three treatment groups in this trial. Because we do not know which treatment is most effective, you will be allocated to one of the three treatment groups by a process called randomisation, which means that the chance of you getting either of the three treatments is the same. Until the randomisation is done, neither the doctor nor you will know which treatment you will receive. Depending on which treatment group you are allocated, you will receive an oral drug for 28 days (miltefosine) or an injection by needle and fluid drip into a vein of your arm over 2 hours (AmBisome) followed by either: 1) 10 days of another daily injection (SSG) or 2) for 10 days of oral treatment (miltefosine).

You will be admitted to the hospital ward for 28 days after the treatment starts. After you go home, we shall want you to return twice for follow-up visits at around 1 and 6-

months after the end of treatment. These follow up visits are very important to make sure you are completely better and that the drug we gave you has worked. This may mean absence from work on those days.

Known side effects of these drugs include: stomach, chest and back pain; shivering and sweating; nausea and vomiting; diarrhoea; skin rashes and feeling tired. Less commonly the drugs may bring damage to the kidneys and liver. During the treatment we shall carefully monitor you. Throughout the time you are in hospital, we will regularly assess your progress by means of blood and urine tests, and heart tracings. We will also be taking additional blood samples to measure concentrations of one of the drugs in your blood as well as to monitor your response to treatment. A total of 7.5 ml of blood will be taken at the beginning of the trial. Samples will be taken again 3 days after treatment and at each weekly assessment during treatment and at follow up schedules to a total of around 60 mls. We shall need to collect tissue from your lymph node/ spleen (Kenya only) or bone marrow to determine if the drug is killing the parasites. This will be done 28 days after you started treatment and at the 6-month follow up visit; and at any time during the follow-up period if the treatment has failed to cure you from the disease. The collection of tissue from bone marrow or lymph nodes/ spleen (Kenya only) will also be done during the initial diagnosis - as this is the standard procedure to confirm the diagnosis of VL. If it is necessary to do a bone marrow test we will give you a local anaesthetic to reduce the pain of this procedure.

In some patients, there might be failure of treatment using the study drugs. If this happens, you will receive a full dose of the medicine called AmBisome®. In those patients who do not respond to a full dose of AmBisome® or suffer from serious side effects, Sodium Stibogluconate (SSG) will be given.

Benefits

The main benefit of participation in this study is that you will be managed closely for the disease. If the study is successful, it means that an alternative shorter treatment will be available for this disease, which will benefit your community and may reduce the likelihood of other people getting the disease.

Confidentiality

At the end of the study, we plan to write a report about the results of the study. The reports will not bear any information relating to you personally e.g. your name or where you live. We are, thus, asking for your permission to use the test results for writing a report. In addition, authorised medical staff, clinical monitors of DNDi (the sponsor of this trial), auditors or representatives of ethics committees or regulatory authorities may wish to inspect your hospital and trial records.

Right to refuse or withdraw

You do not have to take part in this study; your participation is voluntary. If you decide not to take part, you will still be treated at this centre at no cost to you. If you decide to take part and then change your mind later, you may do so, at any time, without losing any of your rights as a patient. It is also possible that we may decide to withdraw you from the trial if we believe it is in your best interests, in which case you will continue to receive the usual treatment for visceral leishmaniasis until you are better.

DNDi may also decide to stop the trial for valid reasons. In this event, we will continue to treat you until you are better. In the event that you suffer an injury or illness related to participating in this trial, DNDi will pay all costs relating to treatment of the injury or illness.

During the course of the trial, if new information becomes available about the treatment, we will tell you about it and discuss whether you want to or should continue in the study. If you decide to continue in the study you will be asked to sign an updated consent form. If you decide not to carry on, we will make the necessary arrangements for your care to continue.

Please note that you will not receive any money for your participation in the trial. However, we will provide you with food and pay your travel expenses to attend the hospital for treatment and hospital follow up visits at 1 and 6 months after treatment.

If you agree to participate in the study, we will ask you to read and sign the consent form.

Do you have any questions?

Patient information for HIV testing

As we have explained to you, you have visceral leishmaniasis; and you have also been invited to participate in the study we have explained in detail. For participation in the study, we need you to be tested for another infection. It is a test for HIV infection. If you happen to be HIV positive, you will not be able to participate in the proposed trial. We will have to treat you with SSG – the standard treatment for visceral leishmaniasis.

We advise you to consider being tested for HIV. Once you are tested, it will be beneficial for you to know the test results, both for your own well being and also for your family, friends and other persons living with you. If you agree to be tested, a specially trained counsellor will hold confidential discussions with you before and after the test, who will then inform you of the test results. If you happen to be HIV positive, we will first treat you for visceral leishmaniasis, and then ensure you are treated for the HIV infection if you fulfil the national criteria for anti-retroviral therapy. You will be provided with anti-retroviral therapy as required by national guidelines by the HIV national control programme at no cost to you. This anti-retroviral treatment will be provided to you for at least 3 years during the study, and further arrangements will be made to make the treatment available throughout your life.

If you do not wish to be tested for HIV, you will not be able to take part in the current trial, but we shall still treat you for visceral leishmaniasis.

PART 2. CONSENT FORM

CONSENT FORM FOR INCLUSION IN THE TRIAL (for signatures)

I, the undersigned, confirm that, as I give consent to participate in the study, it is with a clear understanding of the objectives and conditions of the study and with the recognition of my right to withdraw from the study if I change my mind.

I ……………………….. do hereby give consent to Dr …………………………………… to include me in the proposed research and the treatment. I have been given the necessary information and understand that there might be some risks involved in the treatment or trial procedures.

I have also been assured that I can withdraw my consent at any time without penalty or loss of the benefit of treatment. The study has been explained to me in the language I understand.

Name of Patient: ____

Patient's Signature: __________________________________

Date:__________________

Name of Doctor: _______________________________________

Doctor's Signature: __________________________________

Date:__________________

Name of Witness: ______________________________________

Signature of Witness: ________________________________

Date:__________________

CONSENT FORM FOR HIV TESTING, for those patients with age 18 and above

I, the undersigned, confirm that, as I give consent to HIV testing, it is with a clear understanding of the objectives of HIV testing in this study, the availability of counselling services, the confidentiality of the test results; and in the case that I am HIV positive, the possibility of receiving anti-retroviral therapy should I fulfil the criteria set by the national guidelines.

I,____________________, hereby give consent to Dr __________________ to perform this test. I have been given the necessary information in a language that I understand.

Name of patient: _____________________________________

Patient/Parent/Guardian Signature: _____________________

Date: _________________

Name of Doctor: ____________________________________

Doctor's Signature: _________________________________

Date: _________________

Witness: Name: ____________________________

Signature: ___________________

Date:__________________

TITLE:

"A phase II randomized, parallel arm, open-labeled clinical trial to assess the safety and efficacy of the combination of sodium stibogluconate plus single dose AmBisome®, Miltefosine plus single dose AmBisome® and Miltefosine alone for the treatment of primary visceral leishmaniasis in Eastern Africa. "

EXAMPLE PATIENT INFORMATION AND CONSENT FORM

Form 2:	***For patients under 18 and minors***

SPONSOR: Drugs for Neglected Diseases initiative (DNDi), Geneva, Switzerland

Contact persons

1. Dr. Ahmed Musa
   Dooka Hospital

2. Dr Monique Wasunna
   KEMRI

3. [Chairs, National Ethics Review Committee, and Institutional Ethics Review Committee, (Sudan and Kenya)]

Important notice!

This patient information and consent form is to be read in the language that the patient understands. Therefore, please ask the patient for the preferred language. This form is available in English and Arabic.

PART 1. INFORMATION SHEET

Principal Investigators

Prof E Khalil Institute of Endemic Diseases, University of Khartoum, Sudan

Dr Ahmed Musa Institute of Endemic Diseases, University of Khartoum, Sudan

Dr Monique Wasunna Kenya Medical Research Institute

Sponsor: Drugs for Neglected Diseases *initiative* (DNDi), Geneva, Switzerland

Introduction

We are studying visceral leishmaniasis (also called kala azar) - a fatal disease if untreated, which is common in our country. The tests your child (ward) has had performed confirm that he/she is suffering from this disease.

We are studying different ways of giving the 3 drugs called AmBisome®, sodium stibogluconate and miltefosine to treat this disease, and we would like the child to participate in this study. These drugs alone have been shown to be very effective in treating visceral leishmaniasis either in many countries. In this study, we plan to compare different ways of giving these drugs. Firstly we want to establish that one of the drugs, miltefosine works in the same way it has been demonstrated to in other countries like India. Secondly, we intend to give the drugs in combination with each other- for instance by giving AmBisome® and sodium stibogluconate and AmBisome® and miltefosine together for 10 days. At the end of this trial, we hope that a treatment regimen, which is safe, effective and shorter than the usual treatment duration will be available. This trial is expected to last 18 months, and we intend to enrol up to 189 patients. With your permission, we would like to include the child in this trial. The child's participation will be for 7 months. If you do not give consent to participate in this study the child will be treated with sodium stibogluconate – which is the standard drug for treatment of the disease in this country.

Procedures during the trial

As explained above, there are three treatment groups in this trial. Because we do not know which treatment is most effective, the child will be allocated to one of the three treatment groups by a process called randomisation, which means that the chance of he/she getting either of the three treatments is the same. Until the randomisation is done, neither the doctor nor you will know which treatment he/she will receive. Depending on which treatment group he/she are allocated, he/she will receive an oral drug for 28 days (miltefosine) or an injection by needle and fluid drip into a vein of his/her arm over 2 hours (AmBisome) followed by either: 1) 10 days of another daily injection (SSG) or 2) for 10 days of oral treatment (miltefosine).

The child will be admitted to the hospital ward for 28 days after the treatment starts. After the child goes home, we shall want him/her to return twice for follow-up visits at around 1 and 6-months after the end of treatment. These follow up visits are very

important to make sure the child is completely better and that the drug we gave has worked. For school children, this will mean absence from school on those days.

Known side effects of these drugs include: stomach, chest and back pain; shivering and sweating; nausea and vomiting; diarrhoea; skin rashes and feeling tired. Less commonly the drugs may bring damage to the kidneys and liver. During the treatment we shall carefully monitor the child. Throughout the time the child is in hospital, we will regularly assess his/her progress by means of blood and urine tests, and heart tracings. We will also be taking additional blood samples to measure concentrations of one of the drugs in your blood as well as to monitor your response to treatment. A total of 7.5 ml of blood will be taken at the beginning of the trial. Samples will be taken again 3 days after treatment and at each weekly assessment during treatment and at follow up schedules to a total of around 55 mls. We shall need to collect tissue from the lymph node/ spleen (Kenya only) or bone marrow to determine if the drug is killing the parasites. This will be done 28 days after the first treatment and at the 6-month follow up visit; and at any time during the follow-up period if the treatment has failed to cure the child from the disease. The collection of tissue from bone marrow or lymph nodes/ spleen (Kenya only) will also be done during the initial diagnosis - as this is the standard procedure to confirm the diagnosis of VL. If it is necessary to do a bone marrow test we will give him/her a local anaesthetic to reduce the pain of this procedure.

In some patients, there might be failure of treatment using the study drugs. If this happens, the child will receive a full dose of the medicine called AmBisome®. In those patients who do not respond to a full dose of AmBisome® or suffer from serious side effects, Sodium Stibogluconate (SSG) will be given.

Benefits

The main benefit of participation in the study is that the child will be managed closely for the disease. If the study is successful, it means that an alternative shorter treatment will be available for this disease, which will benefit your community and may reduce the likelihood of other people getting the disease.

Confidentiality

At the end of the study, we plan to write a report about the results of the study. The reports will not bear any information relating to the child's identity e.g. his/her name or where he/she lives. We are asking for your permission to use the test results for writing a report. In addition, authorised medical staff, clinical monitors of DNDi (the sponsor of this trial), auditors, members of ethical committees or regulatory authorities may wish to inspect the child's hospital and trial records.

Right to refuse or withdraw

It is not obligatory for the child to take part in this study; his/her participation is voluntary. If the child decides not to take part, he/she will be treated at this centre at no cost to you. If the child decides to participate and then changes his/her mind later, he/she may do so, at any time, without losing any of his/her rights as a patient.

It is also possible that we may decide to withdraw the child from the trial if we believe

it is in his/her best interests, in which case he/she will continue to receive the usual treatment for visceral leishmaniasis until he/she is better.

DNDi may also decide to stop the trial for valid reasons. In this event, we will continue to treat the child until he/she is better. In the event that he/she suffers an injury or illness related to participating in this trial, DNDi will pay all costs relating to treatment of the injury or illness.

During the course of the trial, if new information becomes available about the treatment, we will tell you about it and discuss if your child (ward) wants to or should continue in the study. If the child decides to continue in the study you will be asked to sign an updated consent form. If the child decides not to carry on, we will make the necessary arrangements for his/her care to continue.

Please note that you (and the child) will not receive any money for participation in the trial. However, we will provide food and pay you and the child's travel expenses to attend the hospital for treatment and hospital follow up visits at 1 and 6 months after treatment.

If you (and the child) agree to participate in the study, we will ask you to read and sign the consent form.

Do you have any questions?

Patient information for HIV testing

As we have explained to you, your child (ward) has visceral leishmaniasis; and he/she has also been invited to participate in the study we have explained in detail. For participation in this study, we need the child to be tested for another infection. It is a test for HIV infection. If the child happens to be HIV positive, he/she will not be able to participate in the proposed trial and we will have to treat him/her with SSG – the standard treatment for visceral leishmaniasis.

We advise you to consider testing the child for HIV. Once the child is tested, it will be beneficial for both of you to know the test results, both for the child's own well being and also for your family, friends and other persons living with you. If you agree to the test being conducted, a specially trained counsellor will hold confidential discussions with you (and the child) before and after the test, which will then inform you of the test results. If the child happens to be HIV positive, we will first treat him/her for visceral leishmaniasis, and then ensure him/her are treated for the HIV infection if he/she fulfils the national criteria for anti-retroviral therapy. He/she will be provided with anti-retroviral therapy as required by national guidelines by the HIV national control programme, at no cost to you. This anti-retroviral treatment will be provided to him/her for at least 3 years during the study, and further arrangements will be made to make the treatment available throughout his/her life.

If you do not wish the child to be tested for HIV, he/she will not be able to take part in the current trial, but we shall still treat him/her for visceral leishmaniasis .

PART 2. CONSENT FORM

CONSENT FOR INCLUSION IN THE TRIAL (MINORS UNDER 18 YRS)

I Mr/Mrs ________________________________ being a person aged 18 years and above, and being the Parent/Lawful guardian of __________________________ hereby give my consent to Dr_____________________ to include my child/ward in the intended research as explained and understood by me. I have understood the implications, risks and immediate benefits of the tests and the treatment.

I give consent for the tests to be carried, and trial treatment to be given to my child/ward.

I understand that I have the right to withdraw my child/ward from the research at any time, for any reason without losing any of his/her rights as a patient.

In case of withdrawal, I understand that the doctor will continue to take care of my child/ward in the same way as any other patient.

All the above conditions have been explained to me in the language, which I understand well.

Parent/Guardian’s full name ___________________________________

Parent/Guardian’s signature ___________________________________

Date:_________________

Child’s full name ___________________________________

Name of Doctor: _____________________

Doctor’s Signature: ___________________

Date:_________________

Witness’ name: ____

Witness’ signature: ______________________

Date:__________________

MINORS ASSENT FORM FOR INCLUSION IN THE TRIAL (under 18) (for signatures)

I, the undersigned, confirm that, as I give my assent to participate in the study, it is with a clear understanding of the objectives and conditions of the study and with the recognition of my right to withdraw from the study if I change my mind.

I, __________________ do hereby give my assent to Dr __________________ to include me in the proposed research. I have been given the necessary information and understand that there might be some risks involved in the treatment or trial procedures.

I have also been assured that I can withdraw my assent at any time without penalty or loss of the benefit of treatment. The study has been explained to me in the language I understand.

I agree to participate

Name of Minor: _____________________

Minor's Signature: __________________

Date:__________________

Name of Doctor: _____________________

Doctor’s Signature: ___________________

Date:__________________

Witness, Name: ________________________

Signature: ______________________

Date:__________________

Consent Form for HIV testing for children UNDER 18 YRS of age:

I, Mr/Ms ________________________ being a person aged 18 years or over and being the Parent/Lawful guardian of Master/Miss _________________________ give consent to Dr______________________ for doing HIV tests to my child (ward).

I give this consent, with a clear understanding of the objectives of HIV testing in the study, i.e., the availability of counseling services, the confidentiality of the test results, and if my child (ward) is positive for HIV, the possibility of receiving anti-retroviral therapy should he/she fulfill the criteria set by national guidelines.

I understand that I have the right to withdraw him / her from the research at any time, for any reason without penalty or harm. In case of withdrawal, I understand that the physicians will continue to take care of him/her like any other patient. I confirm that I have been given the necessary information in a language that I understand very well.

Parent / Guardian's full name: ____

Parent / Guardian's signature:____

Date:_____________

Child's full name: ____

Name of Doctor: ____

Doctor's Signature: ____

Date: _____________

Witness, Name: __________________________

Signature: ________________

Date: _____________

MINORS ASSENT FORM FOR HIV TESTING (under 18) (for signatures)

I, the undersigned, confirm that, as I give consent to HIV testing, it is with a clear understanding of the objectives of HIV testing in this study, the availability of counselling services, the confidentiality of the test results, and in case that I am HIV positive, the possibility of receiving anti-retroviral therapy should I fulfil the criteria set by the national guidelines.

I,____________________ hereby give consent to Dr __________________ to perform this test.

I have been given the necessary information in a language that I understand.

Name of Minor: _____________________

Minor's Signature: __________________

Date:__________________

Name of Doctor: _________________________

Doctor’s Signature: ___________________

Date: _________________

Witness: Name: ________________________

Signature: __________________

Date:__________________

ADVERSE EVENT GRADING to be used for study (*adapted from CTEP, NCI Guidelines – Jan. 2005/ CTC*)

Table of listings of AEs and AE grades (based on selected vital signs, symptoms/signs from Common terminology criteria for adverse events v3.0(CTCAE)

**Symptoms**	AE Grades 1- 4 (AE grade 5 = Death)			
	1	2	3	4
Allergy / Immunology				
Allergic Reaction/ Hypersensitivity	Transient flushing or rash; drug fever <38°C (104.0°F)	Rash; flushing; urticaria; dyspnea; drug fever ≥38°C (104.0°F)	Symptomatic bronchospasm, with or without urticaria; parenteral medication(s) indicated; allergy-related edema/angioedema; hypotension	Anaphylaxis
Bleeding				
Hemorrhage, GI	Mild, intervention (other than iron supplements) not indicated	Symptomatic and medical intervention or minor cauterization indicated	Transfusion, interventional radiology, endoscopic, or operative intervention indicated; radiation therapy (i.e., hemostasis of bleeding site)	Life-threatening; major urgent intervention indicated
Cardiac				
Prolonged QTc intehrval	QTc > 0.45 – 0.47 sec	QTc > 0.47 – 0.50 sec; ≥ 0.06 sec above baseline	QTc > 0.50 sec	QTc > 0.50 sec; life-threatening signs or symptoms (e.g., arrhythmia, CHF, hypotension, shock, syncope); Torsade de pointes
Ventricular arrhythmia	Asymptomatic, no intervention indicated	Non-urgent medical intervention indicated	Symptomatic and incompletely controlled medically or controlled with device (e.g., defibrillator)	Life-threatening (e.g., arrhythmia associated with CHF, hypotension, syncope, shock)
Hypertension	Asymptomatic, transient (<24 hrs) increase by > 20 mmHg (diastolic) or to > 150/100 if previously WNL; intervention not indicated.<br><br>Pediatric:<br>Asymptomatic, transient (<24 hrs) BP increase by >ULN; intervention not indicated	Recurrent or persistent (≥24 hrs) or symptomatic increase by > 20 mmHg (diastolic) or to > 150/100 if previously WNL; monotherapy may be indicated.<br><br>Pediatric:<br>Recurrent or persistent (≥24 hrs) BP >ULN; monotherapy may be indicated	Requiring more than one drug or more intensive therapy than previously<br><br>Pediatric<br>Same as adult	Life-threatening consequences (e.g., hypertensive crisis)<br><br>Pediatric<br>Same as adult
Hypotension	Changes, intervention not indicated	Brief (<24 hrs) fluid replacement or other therapy; no physiologic consequences	Sustained (≥24 hrs) therapy, resolves without persisting physiologic consequences	Shock (e.g., academia; impairment of vital organ function)
Constitutional symptoms				
Fever	38.0 – 39.0°C (100.4 – 102.2°F)	>39.0 – 40.0°C (102.3 – 104.0°F)	>40.0°C (>104.0°F) for ≤24 hrs	>40.0°C(>104.0°F) for >24 hrs
Rigors/chills	Mild	Moderate, narcotics	Severe or prolonged, not	-

Symptoms	AE Grades 1- 4 (AE grade 5 = Death)			
	1	2	3	4
		indicated	responsive to narcotics	
Weight loss	5 to <10% from base line; intervention not indicated	10 - <20% from baseline; nutritional support indicated	>20% from baseline; tube feeding or TPN indicated	-
Dermatology Skin				
Injection site reaction / extravasation changes	Pain, itching ; erythema	Pain or swelling with inflammation or phlebitis	Ulceration or necrosis that is severe; operative intervention indicated	-
Rash	Macular or popular eruption or erythema without associated symptoms	Macular or popular eruption or erythema with pruritus or other associated symptoms; localized desquamation or other lesions covering <50% of body surface area (BSA)	Severe, generalized erythroderma or macular, papular or vesicular eruption; desquamation covering >50% BSA	Generalized exfoliative, ulcerative, or bullous dermatitis
Gastrointestinal;				
Anorexia	Loss of appetite, without alteration in eating habits	Oral intake altered without significant weight loss or malnutrition; oral nutritional supplements indicated	Weight loss / malnutrition (inadequate caloric / fluid intake); treatments indicated (IV fluids, tube feedings, TPN)	Life threatening consequences
Diarrhoea	Increase of <4 stools per dayover baseline; mild increase in ostomy output	Increase 4-6 stools /day over baseline. IV fluid indicated <24hrs; moderate increase in ostomy output; not interfering with ADL	Increase ≥7 stools /day over baseline; incontinence; IV fluid indicated ≥24 hrs; hospitalization; severe increase in ostomy output; interfering with ADL	Life threatening consequences (eg Hemodynamic collapse)
Dehydration	Increased oral fluids indicated; dry mucous membranes; diminished skin turgor	IV fluids indicated <24 hrs	IV fluids indicated ≥24 hrs	Life-threatening (e.g. hemodynamic collapse)
Nausea	Loss of appetite without alteration in eating habits	Oral intake decreased without significant weight loss, dehydration or malnutrition; IV fluids indicated < 24 hrs	Inadequate oral caloric or fluid intake; IV fluids, tube feeding, or TPN indicated ≥ 24 hrs	Life threatening consequences
Vomiting	1 episode in 24 hrs	2-5 episodes in 24 hrs; IV fluid indicated < 24 hrs	≥ 6 episodes in 24 hrs; IV fluids, or TPN indicated for ≥ 24 hrs	Life threatening consequences
Hepatobiliary / Pancreas				
Pancreatitis	Asymptomatic. Enzyme elevation and/or radiographic findings	Symptomatic, medical intervention indicated	Interventional radiology or operative intervention indicated	Life-threatening consequences (e.g., circulatory failure, hemorrhage, sepsis)
Liver dysfunction / failure	-	Jaundice	Asterixis	Encephalopathy or coma
Renal Genitourinary				
Renal failure	-	-	Chronic dialysis not indicated	Chronic dialysis or renal transplant indicated

Symptoms	AE Grades 1- 4 (AE grade 5 = Death)			
	1	2	3	4
Infection				
Infection[1]	-	Localized, local intervention indicated	IV antibiotic, antifungal, or anti-viral intervention indicated; interventional radiology or operative intervention indicated	Life-threatening consequences (e.g., septic shock, hypotension, acidosis, necrosis)
Infection<br><br>(with normal ANC or grade 1 or 2 neutrophils)	-	Localized, local intervention indicated	IV antibiotic, antifungal, or anti-viral intervention indicated; interventional radiology or operative intervention indicated	Life-threatening consequences (e.g., septic shock, hypotension, acidosis, necrosis)
Febrile neutropenia[2]	-	-	Present	Life-threatening consequences (e.g., septic shock, hypotension, acidosis, necrosis)
Viral hepatitis	Present; transaminases and liver function normal	Transaminases abnormal, liver function normal	Symptomatic liver dysfunction; fibrosis by biopsy; compensated cirrhosis	Decompensated liver function (e.g., ascites, coagulopathy, encephalopathy, coma
Infection Other	mild	moderate	severe	Life-threatening, disabling
Pain				
Pain (site to be specified)	Mild pain not interfering with function	Moderate pain; pain or analgesics interfering with function but not interfering with ADL	Severe pain; pain or analgesics severely interfering with ADL	Disabling
Pulmonary Upper respiratory				
Bronchospasm, wheezing	Asymptomatic	Symptomatic, not interfering with function	Symptomatic interfering with function	Life-threatening
Cough	Symptomatic non-narcotic medication only indicated	Symptomatic and narcotic Medication indicated	Symptomatic and significantly interfering with sleep or ADL	-
Hypoxia	-	Decreased O2 saturation with exercise (e.g. pulse oximeter<br>< 88%); intermittent supplemental oxygen	Decreased O2 saturation at rest; continuous oxygen indicated	Life-threatening; intubation or ventilation indicated
Laboratory				
Hgb (Decrease)	<LLN – 10.0 g/dL	<10.0 – 8.0g/dL	<8.0 – 6.5g/dL	<6.5g/dL
WBC counts (Decrease)	<LLN - 3,000/mm^3	<3,000 - 2,000/mm^3	<2,000 - 1,000/mm^3	< 1000/mm^3
Platelet counts (Decrease)	<LLN - 75,000/mm^3	<75,000 - 50,000/mm^3	<50,000 - 25,000/mm^3	< 25,000/mm^3
Albumin (Decrease)	< LLN – 3.0 g/dL	< 3.0 – 2.0 g/dLg/dL	< 2.0 g/dL	-
Alkaline phosphatase (Elevation)	> ULN – 2.5x ULN	> 2.5 – 5.0x ULN	> 5.0 – 20.0x ULN	> 20.0x ULN
ALT/SGPT (Elevation)	> ULN – 2.5x ULN	> 2.5 – 5.0x ULN	> 5.0 – 20.0x ULN	> 20.0x ULN

1 Documented clinically or microbiologically with grade 3 or 4 neutrophils; ANC <1.0x10^9/L

2 Febrile Neutropenia (fever of unknown origin without clinically or microbiologically documented infection); ANC <1.0x10^9/L; and fever $\geq$38.5^{o}C]

**Symptoms**	AE Grades 1- 4 (AE grade 5 = Death)			
	1	2	3	4
AST/SGOT (Elevation)	> ULN – 2.5x ULN	> 2.5 – 5.0x ULN	> 5.0 – 20.0x ULN	> 20.0x ULN
Amylase (Elevation)	> ULN – 1.5x ULN	> 1.5 – 2.0x ULN	> 2.0 – 5.0x ULN	> 5.0x ULN
Bilirubin (Elevation)	>ULN – 1.5x ULN	> 1.5 – 3.0x ULN	>3.0 – 10.0x ULN	> 10.0x ULN
Creatinine (Elevation)	>ULN – 1.5x ULN	> 1.5 – 3.0x ULN	>3.0 – 6.0x ULN	> 6.0x ULN
Magnesium (Increase)	>ULN – 3.0 mg/dL	-	3.0 – 8.0 mg/dL	>8.0 mg/dL
Magnesium (Decrease)	<LLN – 1.2 mg/dL	<1.2 – 0.9 mg/dL	<0.9 – 0.7 mg/dL	<0.7 mg/dL
Potassium (Decrease)	<LLN – 3.0mmol/L	-	< 3.0 – 2.5 mmol/L	<2.5 mmol/L
Sodium (Increase)	>ULN – 150 mmol/L	>150 – 155 mmol/L	>155 – 160 mmol/L	>160 mmol/L
Sodium (Decrease)	<LLN – 130 mmol/L	-	<130 – 120 mmol/L	<120 mmol/L
Proteinuria (Elevation)	0.15 – 1.0 g/24 hr (1+)	>1.0 – 3.5 g/24 hr (2/3+)	>3.5g/24 hr (4+)	Nephrotic syndrome

Changes introduced in protocol version

None at present

Budget (in Euros)

Budget lines	Year 1	Year 2	Year 3	Total
Purchase & logistics	25000	40000	10000	75000
Training	10000	20000	2000	32000
Clinical/ patient costs	129350	1000000	75000	1204350
Personnel costs	57145	100000	30000	187145
Administrative costs	5000	7000	3000	15000
Communication costs	30000	50000	10000	90000
Total	256495	1217000	130000	1 603 495
